# Hydroxyurea blunts mitochondrial energy metabolism and osteoblast and osteoclast differentiation exacerbating trabecular bone loss in sickle cell mice

**DOI:** 10.1038/s41419-024-07296-z

**Published:** 2024-12-18

**Authors:** Ashish Kumar Tripathi, Sadaf Dabeer, Jun Song, Tatyana Vikulina, Susanne Roser-Page, Jessica A. Alvarez, David. R. Archer, M. Neale Weitzmann

**Affiliations:** 1 Division of Endocrinology, Metabolism, and Lipids, Department of Medicine, Emory University School of Medicine, Atlanta, Georgia USA; 2 The Atlanta Department of Veterans Affairs Medical Center, Decatur, GA USA; 3 Department of Endocrinology, Guang’anmen Hospital, China Academy of Chinese Medical Sciences, Beijing, China; 4 Aflac Cancer and Blood Disorder Center, Children’s Healthcare of Atlanta, Emory University, Atlanta, GA USA

**Keywords:** Osteoporosis, Drug safety

## Abstract

Sickle cell disease (SCD) is a severe hematological disorder characterized by erythrocyte sickling that causes significant morbidity and mortality. Skeletal complications of SCD include a high incidence of bone loss, especially in vertebrae, leading to fragility fractures that contribute to disease burden. Whether hydroxyurea (HU), a front-line therapy for SCD ameliorates bone disease has not been established. To investigate HU action on SCD-related vertebral defects, we used HU-treated “Townes” mice, an SCD animal model and performed high-resolution micro-computed tomography (µCT) imaging to resolve bone volume and micro-architectural structure of cortical and trabecular bone, the two major compartments contributing to bone mass and strength. Our data revealed that cortical bone was significantly diminished in the vertebrae of skeletally mature (representing adults) and immature (representing children) SCD mice, while only mature mice lost trabecular bone mass. Administration of HU ameliorated cortical bone loss in mature SCD mice, but paradoxically promoted trabecular bone decline in both groups. We further investigated the mechanisms of HU action in wild-type C57BL6/J mice. HU caused dose-dependent trabecular bone loss due to diminished osteoclast and osteoblast function, indicative of a low bone turnover state. Mechanistic investigations in vitro revealed that HU impeded osteoblast-progenitor proliferation and early differentiation, and diminished osteoclastogenic cytokine production, blunting osteoclast formation as well as the activity of mature osteoclasts. HU further, suppressed mitochondrial, but not glycolytic energy metabolism in both differentiating osteoblasts and differentiated osteoclasts. Collectively, these findings reveal that despite ameliorating cortical bone loss, HU inhibits trabecular bone formation and resorption, by suppressing mitochondrial energy metabolism and blunting the differentiation and/or activity of osteoblasts and osteoclasts. Together HU drives a low bone turnover state culminating in trabecular bone loss. Further investigation into HU’s impact on bone in SCD patients is warranted for understanding and managing skeletal complications in this population.

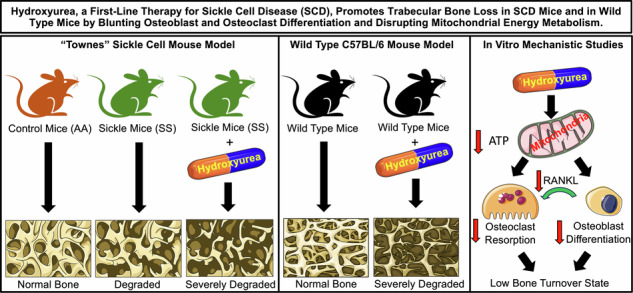

## Introduction

Sickle cell disease (SCD) is an inherited hemolytic disorder stemming from a mutation in the *hemoglobin-β* gene and affects >100,000 people in the U.S. and millions around the world [1].

Hydroxyurea (HU) (hydroxycarbamide) is a first-line pharmacotherapy for SCD [1, 2]. HU promotes fetal hemoglobin production that diminishes deoxygenated sickle globin polymerization clinically reducing disease morbidities [3]. HU is standard-of-care for the most common types of SCD (SS- and Sß^0^-thalassemia) and is prescribed ubiquitously and at a young age, irrespective of SCD severity.

Osteoporosis, a serious skeletal disease, is common in SCD and characterized by significant deficits in bone mineral density (BMD) [4–13] with 66% of patients displaying low BMD at the lumbar spine [4]. Low BMD predisposes patients to fractures and vertebral deformities that contribute to medical costs and poor quality of life in SCD patients [12–15]. Bone damage may also contribute to unremitting bone pain as 80% of SCD patients with chronic bone pain also exhibited low BMD and demonstrated skeletal abnormalities by radiography [13].

SCD and ensuing skeletal abnormalities have been extensively studied in “Townes” mice, a sophisticated humanized SCD mouse model that recapitulates significant features of SCD [16] including bone loss [17–22]. This bone loss has been ascribed to increased bone resorption and decreased bone formation that leads to cortical bone loss and a trend toward trabecular bone decline. Mechanistically, SCD was associated with a decrease in insulin-like growth factor-1 (IGF-1), secondary to diminished short-chain fatty acid production [17, 20]. This bone disease was alleviated by fecal microbiota transplantation from wild-type (WT) mice into SCD mice, which enhanced microbiota production of short-chain fatty acids and restored IGF-1 production [17].

In another bone study in Townes mice, bone loss was found to be sex-dependent with more prevalent trabecular bone changes occurring in female mice, while cortical bone deterioration was observed in both sexes [18].

Another study in SCD mice revealed that under normoxic conditions, bone impairments were related to increased osteoclast and reduced osteoblast activity, while under hypoxic conditions, mimicking vaso-occlusive crises, bone turnover was increased, with elevated osteoclast activity and recruitment, and with elevated production of the pro-inflammatory cytokine IL-6 [23].

Given that HU ameliorates sequelae of SCD, it might be expected to also alleviate SCD-induced burden on the skeleton. However, whether HU is, in fact, beneficial for the growing skeletons of children with SCD or for physiological bone regeneration and repair programs in adults with SCD has not been established.

In the present study, we investigated the effects of HU on bone turnover, structure, and osteoclasts and osteoblasts, using Townes SCD and wild-type (WT) mice. We report that although there is evidence of some beneficial effects on cortical bone, HU causes significant deterioration of the trabecular bone compartment, driven by a decline in osteoclast and osteoblast differentiation and function.

## Results

### HU diminishes trabecular vertebral bone mass in skeletally immature SS mice

**Fig. 1:**
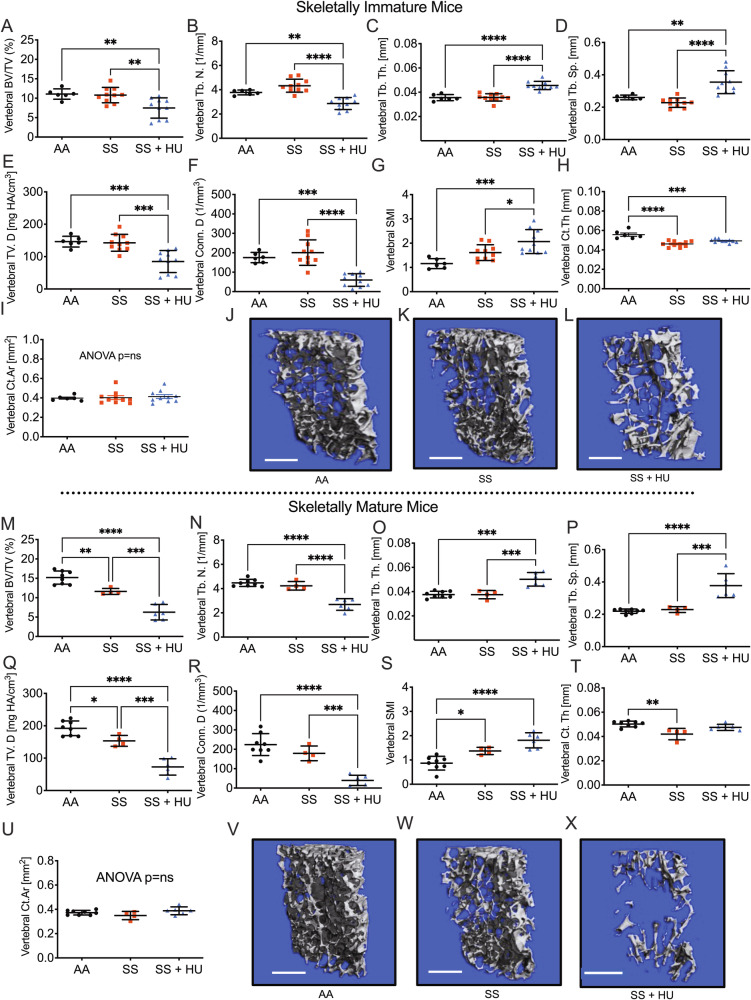
Ex vivo µCT analysis of lumbar vertebrae from skeletally immature and skeletally mature AA control and SS sickle cell disease mice. L3 vertebrae from AA control mice, SS sickle mice, and SS mice treated with HU (40 mg/kg/twice/week) were analyzed by high-resolution (6 µm) µCT and the trabecular and cortical bone compartments segregated for: Skeletally immature male mice (9–11 weeks of age): **A** BV/TV, **B** Tb.N, **C** Tb.Th, **D** Tb.Sp, **E** TV.D, **F** Conn.D, and **G** SMI. Cortical indices: **H** Ct.Th and **I** Ct.Ar. Representative high-resolution (6 µm) 3D visual reconstructions of L3 vertebrae for **J** AA mice, **K** SS mice and **L** SS mice + HU. *n* = 6 AA, 10 SS and 10 SS + HU mice/group. Skeletally mature male mice (15–18 weeks of age): **M** BV/TV, **N** Tb.N, **O** Tb.Th, **P** Tb.Sp, **Q** TV.D, **R** Conn.D, and **S** SMI. Cortical indices: **T** Ct.Th and **U** Ct.Ar. Representative high-resolution (6 µm) 3D visual reconstructions of L3 vertebrae for **V** AA mice, **W** SS mice and **X** SS mice + HU. *n* = 8 AA, 4 SS and 6 SS + HU mice/group. White scale lines represent 500 µm. Data expressed as Mean ± S.D. *p < 0.05, **p < 0.01, ***p < 0.001, ****p < 0.0001. Non-significant comparisons are not shown. Data were analyzed by one-way ANOVA with Tukey post hoc test or Kruskal–Wallis with Dunn’s post hoc test [**I** Ct.Ar and **T** Ct.Th] if nonparametric as assessed by the Shapiro–Wilk normality test. ANOVA not significant (ANOVA *p* = ns).

To model HU on SCD on child vertebrae, skeletally immature (9–11 weeks of age) male “Townes” sickle mice (SS), and age-matched non-sickling controls (AA), were treated for 12 weeks with HU (40 mg/kg/day-2-times/week) and vertebrae imaged by micro-computed tomography (µCT). Trabecular bone volume fraction (BV/TV), the key index of trabecular bone mass, was not significantly different between young SS and AA mice; however, HU caused a significant decline (Fig. 1A). The trabecular structural indices trabecular number (Tb.N), trabecular thickness (Tb.Th), and trabecular separation (Tb.Sp) were not altered in SS relative to AA mice (Fig. 1B–D), however HU treatment significantly decreased Tb.N, while Tb.Sp and Tb.Th were significantly increased (Fig. 1B–D). Trabecular volumetric bone density (TV.D) and connectivity density (Conn.D) were unchanged between SS and AA mice but significantly diminished by HU (Fig. 1E, F). The micro-architectural structure model index (SMI) was unaltered in SS mice, but significantly increased by HU, suggesting compromised trabecular structure (Fig. 1G). Figure 1J–L shows vertebral trabecular reconstructions.

In contrast to trabecular bone, cortical thickness (Ct.Th), but not cortical area (Ct.Ar) declined in SS mice; however, HU treatment had no effect (Fig. 1H, I).

### SCD causes vertebral bone loss that is exacerbated by HU in skeletally mature SS mice

As skeletal maturity occurs ~16–18 weeks of age in mice [24] we examined HU on a mature “remodeling” skeleton using 15–18 weeks of age SCD mice. In contrast to immature SS mice, BV/TV declined significantly in SS vertebrae (Fig. 1M), and HU caused a further significant decline in BV/TV (Fig. 1M). Despite a modest decline in BV/TV, the structural indices Tb.N, Tb.Th and Tb.Sp were not different in SS mice (Fig. 1N–P), although an increase in SMI and a reduction in TV.D, without changes in Conn.D, were observed (Fig. 1Q–S). HU caused significant reductions in Tb.N, TV.D and Conn.D and increases in Tb.Sp and SMI (Fig. 1N, P–S) indicative of diminished bone mass and architectural deterioration, while Tb.Th was increased (Fig. 1O). µCT reconstructions are presented in Fig. 1V–X.

As in young SS mice, Ct.Th, but not Ct.Ar was significantly diminished (Fig. 1T, U). However, HU caused a modest increase in Ct.Th (but not Ct.Ar), thus alleviating, in part, cortical bone loss in SS mice (Fig. 1T, U).

The data suggest that while HU ameliorates in part, cortical bone loss, trabecular bone is further damaged in both skeletally immature and mature mice.

### HU induces dose-dependent declines in vertebral trabecular bone mass in WT mice

To study mechanisms of HU action on the skeleton, we administered HU (5 days/week) to 8-week-old WT female C57BL6/J mice for 30 days at 35 mg/kg/day representing unadjusted weight-based scaling from human dose, 75 mg/kg/day an allometrically (¾-power) scaled dose compensating for increased metabolic rate of the mouse [25] and 150 mg/kg/day, a supra-pharmacological dose to maximize sensitivity for mechanistic investigations.

**Fig. 2:**
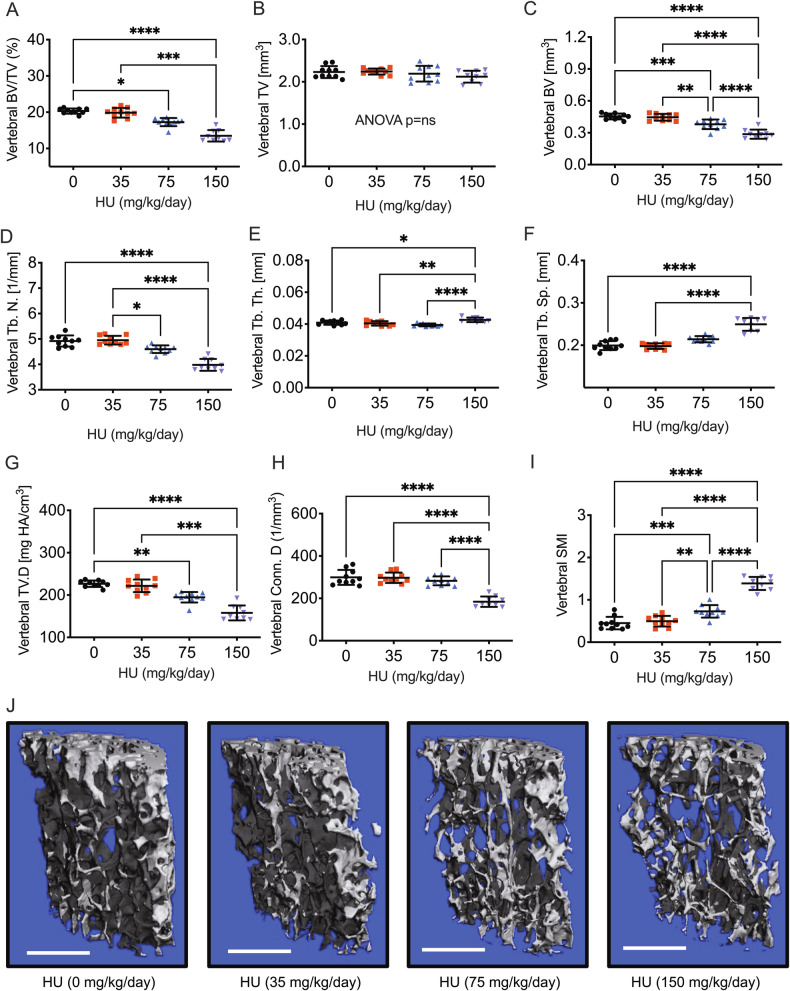
Ex vivo µCT analysis of lumbar vertebrae from WT mice treated with HU. L3 vertebrae from WT female C57BL6/J mice (8 weeks of age) treated for 30 days with HU (35, 75 and 150 mg/kg/week) were analyzed by high-resolution (6 µm) µCT. **A** BV/TV, **B** TV, **C** BV, **D** Tb.N, **E** Tb.Th, **F** Tb.Sp, **G** TV.D, **H** Conn.D, and **I** SMI. **J** Representative high-resolution (6 µm) 3D visual reconstructions of L3 vertebrae. *n* = 10 mice per group for 0, 35, and 75 mg/kg/week and 9 mice/group for 150 mg/kg/week. White scale lines represent 500 µm. Data expressed as Mean ± S.D. *p < 0.05, **p < 0.01, ***p < 0.001, ****p < 0.0001. Non-significant comparisons are not shown. Data analyzed by one-way ANOVA with Tukey post hoc test or Kruskal–Wallis test with Dunn’s post hoc test [**A** BV/TV, **D** Tb.N, **G** TV.D and **I** SMI] if nonparametric as assessed by the Shapiro–Wilk normality test.

As in SS mice, HU caused a significant decline in vertebral BV/TV in WT mice at 75 and 150 mg/kg/day (Fig. 2A). To assess the contributions of the BV/TV component parts bone volume (BV) and tissue volume (TV) were independently quantified. TV a reflection of size, was not impacted by HU at any dose (Fig. 2B) and the effects of HU on BV/TV were fully accounted for by changes in BV alone (Fig. 2C). HU caused a decline in Tb.N (Fig. 2D) and at the highest dose (150 mg/kg/day) also replicated the increase in Tb.Th (Fig. 2E) observed in HU-treated SS mice (Fig. 1C, O). Tb.Sp was significantly elevated at 150 mg/kg/day but not at lower doses (Fig. 2F). TV.D was significantly suppressed, and SMI increased at doses of 75 and 150 mg/kg/day, while Conn.D was only decreased at 150 mg/kg/day (Fig. 2G–I). Reconstructions of the vertebral trabecular compartments are shown in Fig. 2J.

### HU induces dose-dependent declines in femoral trabecular, but not cortical bone mass in WT mice

As SCD can also impact the appendicular skeleton, we further performed µCT analyses of the trabecular-rich femoral distal metaphases and cortical-rich femoral mid-diaphysis of WT HU-treated mice. Outcomes for femur were comparable to the vertebrae, with significant declines in BV/TV at 75 and 150 mg/kg/day, driven by changes in BV. As in vertebrae Tb.N and Conn.D were significantly diminished and Tb.Sp and Tb.Th increased, although changes in Tb.Th were modest and only reached significance at the highest dose. TV.D was not significantly different from control due to high variability, but 150 mg/kg/day of HU was significantly decreased relative to the 35 mg/kg/day HU-treated group. Surprisingly, femoral SMI was not significantly different at any dose (Supplementary Fig. 1A–I). Reconstructions of the femoral trabecular compartment are presented in Supplementary Fig. 1J.

By contrast to trabecular indices, there were no changes in the key cortical indices Ct.Th and Ct.Ar (Supplementary Fig. 2A, B). Reconstructions of the femoral cortical compartment are presented in Supplementary Fig. 2C.

### Dual-energy X-ray absorptiometry (DEXA) fails to detect HU-induced changes in BMD

To assess the utility of DEXA in resolving HU-induced BMD changes, we performed DEXA analysis of WT vehicle and HU (150 mg/kg/day) treated mice after 4 weeks of treatment. DEXA was insensitive in resolving trabecular changes at the total body, femur and even the trabecular-rich lumbar spine (Supplementary Fig. 3A–C).

### HU drives a state of low bone turnover with both diminished bone resorption and bone formation

**Fig. 3:**
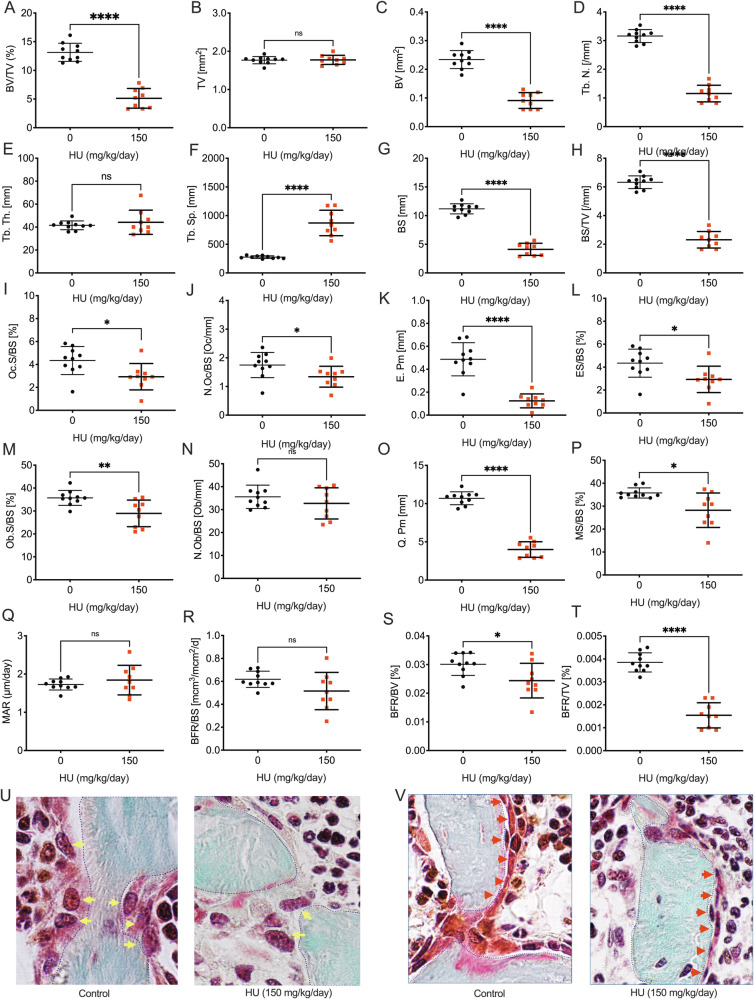
Quantitative bone histomorphometry µCT analysis of lumbar vertebrae from WT mice treated with HU. Histomorphometry was performed on femurs from WT female C57BL6/J mice (8 weeks of age) treated for 30 days with HU (150 mg/kg/week). Bone mass and Structural indices: **A** BV/TV, **B** TV, **C** BV, **D** Tb.N, **E** Tb.Th, **F** Tb.Sp, **G** BS and **H** BS/TV. Osteoclast and bone resorption indices: **I** Oc./BS, **J** N.Oc/BS, **K** E.Pm, **L** ES/BS: Osteoblast and bone formation indices: **M** Ob.S/BS, **N** N.Ob/BS, **O** Q. Pm, **P** MS/BS, **Q** MAR, **R** BFR/BS, **S** BFR/BV and **T** BFR/TV. *n* = 10 mice per group for 0 mg/kg/week and 9 mice/group for 150 mg/kg/week. Data expressed as Mean ± S.D. *p < 0.05, **p < 0.01, ***p < 0.001, ****p < 0.0001. Non-significant comparisons are not shown. Data analyzed by Student’s *t*-test. All data were normally distributed based on the Shapiro–Wilk normality test. Representative histological images of **U** osteoclasts (yellow arrows) and **V** osteoblasts (red arrows) are shown for control and HU (150 mg/kg/day) treated mice. Image magnification 100×.

To study bone turnover at the tissue level, we performed quantitative bone histomorphometry on WT bones treated with vehicle or HU (150 mg/kg/day). Using histomorphometry BV/TV, TV, BV, Tb.N, and Tb.Sp were concordant with µCT outcomes (Fig. 3A–D, F). However, Tb.Th, was unchanged (Fig. 3E) in contrast to µCT where it was increased. Bone surface (BS and BS/TV) were both significantly decreased (Fig. 3G, H).

The osteoclast indices bone surface covered by osteoclasts (Oc.S/BS), the number of osteoclasts per bone surface N.Oc/BS, erosion perimeter (E.Pm), and erosion surface per bone surface (ES/BS) were significantly diminished (Fig. 3I–L) suggesting a decline in osteoclast numbers and resorptive activity.

Bone surface covered by osteoblasts (Ob.S/BS) (Fig. 3M) was significantly diminished, however, total number of osteoblasts per bone surface (N.Ob/BS) was not (Fig. 3N). The quiescent perimeter was significantly decreased by HU, as was the mineralizing surface (MS/BS) (Fig. 3O, P). Mineral apposition rate (MAR) and bone formation rate (BFR/BS) were not significantly decreased (Fig. 3Q, R), however, BFR was significantly diminished by HU after normalizing for BV or TV (Fig. 3S, T). Representative images of osteoclasts and lining osteoblast are shown in Fig. 3U and V respectively. These data suggest diminished osteoblast numbers and bone formation mainly due to diminished bone surfaces, but no changes in the functional activity of pre-existing osteoblasts.

Taken together the data reveal a low bone turnover state with compromised bone resorption and formation in the trabeculae bone compartment.

### HU blunts osteoblast differentiation but does not affect mineralization

**Fig. 4:**
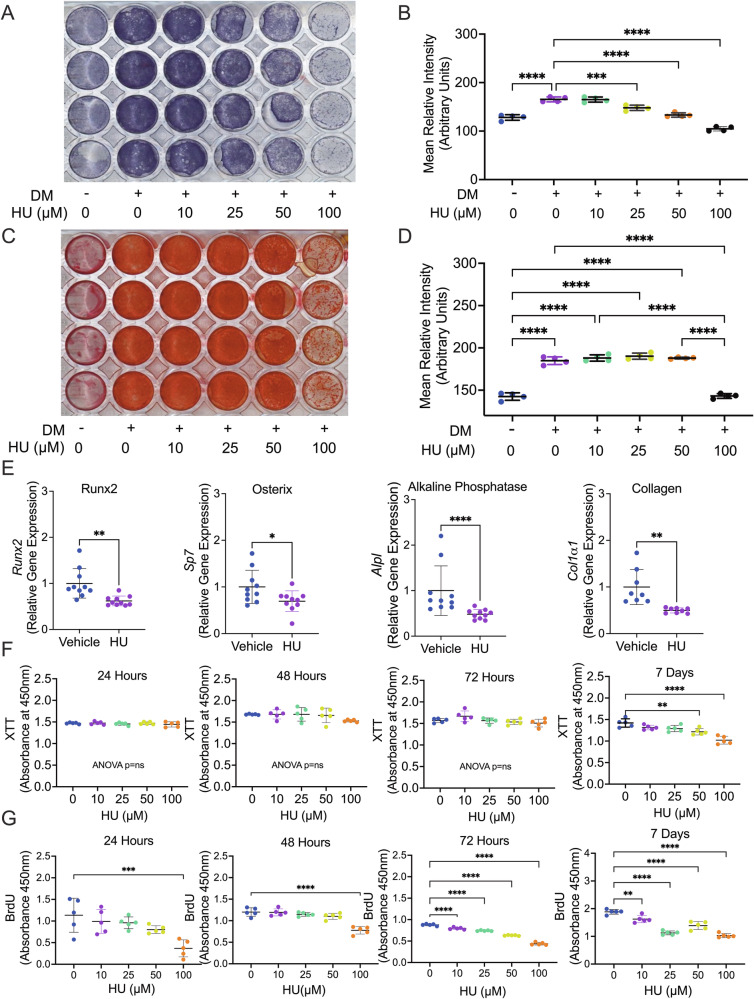
Effects of HU on BMSC proliferation and differentiation into mineralizing osteoblasts in vitro. Highly enriched bone marrow stromal cells (BMSC) isolated from WT mouse bone marrow were cultured in a control medium or differentiation medium (DM) supplemented with ascorbic acid and β-glycerophosphate. Cultures were treated with HU ranging from 10 to 100 µM. **A** Cells plated at 40% confluence were stained for alkaline phosphatase activity at 7 days. **B** Densitometric quantification of alkaline phosphatase staining. **C** Cells plated at 80% confluence and stained for mineral deposition by Alizarin Red S staining at 14 days. **D** Densitometric quantification of Alizarin Red S staining. *n* = 4 wells/group. **E** Osteoblast gene expression was quantified by real-time RT-PCR for Runx2, Osterix, alkaline phosphatase and Collagen type I (Collagen) at 7 days of HU treatment (50). *n* = 10 data points from combined data from 2 independent experiments of *n* = 5. **F** XTT assays at 24 h, 48 h, 72 h and 7 days of HU treatment (50 µM). **G** BrdU ELISA at 24 h, 48 h, 72 h and 7 days of HU (50 µM) treatment. (**F**) and (**G**) are representative data from at least 2 independent experiments of *n* = 5 samples/group. Data expressed as Mean ± S.D. *p < 0.05, **p < 0.01, ***p < 0.001, ****p < 0.0001. Statistical analyses for two groups involved Student’s *t*-test or Mann–Whitney test (**E** Aalkaline phosphatase) if nonparametric as assessed by the Shapiro–Wilk normality test. Multigroup comparisons involved 1-way ANOVA with Tukey post hoc test or Kruskal–Wallis with Dunn’s post hoc test for nonparametric data (**F** XTT at 24 h) as assessed by the Shapiro–Wilk normality test. For panels (**F**) and (**G**) significance is shown relative to control (0 HU). ANOVA not significant (ANOVA *p* = ns).

To investigate HU activity on osteoblasts, we performed in vitro osteoblast differentiation assays using WT bone marrow stromal cells (BMSC) as osteoblast progenitors. BMSC were cultured in differentiation medium (DM) and treated with 10–100 µM HU. After 7 days early differentiation was assessed by alkaline phosphatase staining. Relative to control (non-mineralizing) medium, DM-treated BMSC produced significantly more alkaline phosphatase activity. HU suppressed alkaline phosphatase at ≥25 µM (Fig. 4A, B) suggesting HU impedes BMSC proliferation and early osteoblast differentiation.

To study mineralization, osteoblasts were pre-differentiated before the addition of HU. Mineralization was assessed after 14 days by Alizarin red S staining for mineral deposition. HU had no effect at ≤50 µM, but impeded mineral deposition at 100 µM, likely due to toxicity (Fig. 4C, D).

The data suggest that HU blunts proliferation and early differentiation of osteoblasts, but not late differentiation/mineralization.

### HU blunts expression of osteoblast genes

HU (50 µM) action on expression of osteoblast transcription factors/gene products was determined at 7 days. Runx2, Osterix, Alkaline phosphatase and Collagen Type I were significantly diminished (Fig. 4E), suggesting that HU impedes early differentiation of osteoblasts.

### HU blunts proliferation of BMSC

To determine if HU impedes proliferation of BMSC, delaying differentiation, we performed XTT assays on BMSC at 24 h, 48 h, 72 h and 7 days. HU had no significant effect at doses up to 100 µM for up to 48 h, however, there was a significant decline by 7 days at doses ≥50 µM (Fig. 4F).

As XTT lacks specificity for proliferation, we performed BrdU assays. BrdU was significantly diminished at 100 µM at all time points, and by 72 h, significant declines in proliferation were observed at concentrations as low as 10 µM HU. The data suggest that HU impedes BMSC proliferation and expansion.

To assess HU toxicity, we performed Annexin V/propidium iodide flow cytometry using BMSC exposed to HU for 7 days at 50 µM and 100 µM. No decline in cell viability or increase in apoptosis or necrosis was observed at HU doses of 50 µM (Supplementary Fig. 6A), although a small but significant decrease in cell viability and increase in apoptosis and necrosis was observed in BMSC at 100 µM.

### HU impedes bone resorption and blunts osteoclastogenic cytokine production (RANKL) by BMSC

**Fig. 5:**
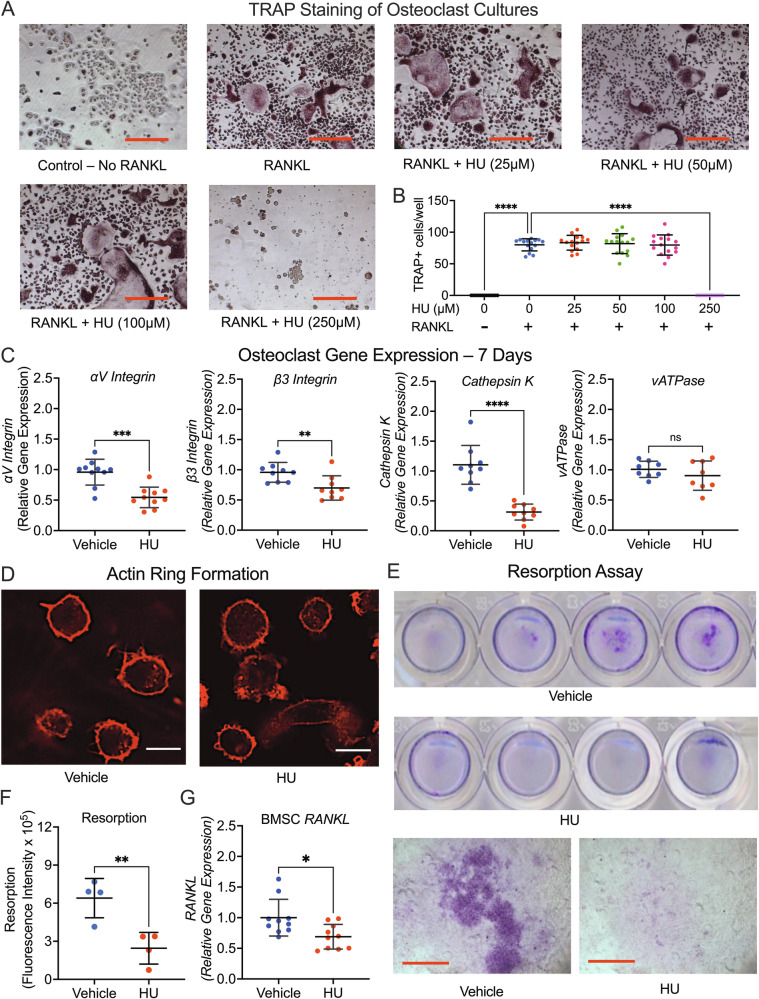
Effect of HU on osteoclastogenesis and resorption in vitro. Purified monocytes isolated from WT mouse spleen were cultured with M-CSF, with and without RANKL, plus HU (0–250 µM). After 7 days, cultures were TRAP stained and osteoclasts quantified multinucleated cells (≥3 nuclei). **A** Photomicrographs of representative osteoclast cultures. Red scale bars represent 50 µm. **B** Osteoclast numbers were quantified from multiple cells combined from 3 independent experiments of *n* = 5 wells/group each. **C** Osteoclast gene expression was quantified by real-time RT-PCR for αV integrin subunit, β3 integrin subunit, Cathepsin K and vATPase at 7 days of HU (50 µM) treatment. *n* = 10 data points from combined data from 2 independent experiments of *n* = 5/group each. **D** Photomicrographs (60× magnification) of actin ring formation from in situ immunohistochemistry. White scale bars represent 20 µm. **E** Resorption assays of HU (50 µM) and vehicle-treated toluidine blue stained wells (*n* = 4 wells/group) and representative photomicrographs of resorption (40× magnification) below. Red scale bars represent 20 µm. **F** Quantification of resorption following exposure to HU (50 µM). **G** *RANKL* expression quantified by RT-PCR in vehicle and HU (50 µM) treated BMSC. Combined data from 2 independent experiments of *n* = 5/group each. Data expressed as Mean ± S.D. *p < 0.05, **p < 0.01, ***p < 0.001, ****p < 0.0001. Statistical analyses for two groups involved Student’s *t*-test. Multigroup comparisons involved 1-way ANOVA with Tukey post-test or Kruskal–Wallis with Dunn’s post-test for nonparametric data (**B**) as assessed by the Shapiro–Wilk normality test. For panel (**B**), significance is shown relative to control (RANKL treated, HU (0 µM) group).

To explore effects of HU on osteoclastogenesis, we performed in vitro assays. Monocytes were treated with RANKL and/or M-CSF and HU (10–250 µM) for 7 days and TRAP-positive multinucleated cells quantified. HU did not affect osteoclastogenesis up to 100 µM, but at ≥250 µM osteoclastogenesis was inhibited, likely due to cytotoxicity (Fig. 5A, B). HU toxicity was further examined by Annexin V/propidium iodide flow cytometry using monocytes exposed to HU for 7 days. No toxic effect was observed at 50 µM for any index, however a small but statistically significant increase in necrotic cells (but not in viability or apoptosis) was observed at 100 µM (Supplementary Fig. 6B).

Key osteoclast genes αV and β3 integrin subunits, cathepsin K and vATPase were not affected by 50 µM HU at 3 and 5 days of treatment (Supplementary Fig. 4), but by 7 days, all gene expression except for vATPase was significantly diminished (Fig. 5C).

Actin ring formation, a key step in the formation of the sealing zone necessary for osteoclast resorption was not affected by 50 µM HU treatment (Fig. 5D).

As osteoclastogenesis and bone resorption are independent processes, we performed an in vitro osteoclast resorption assay. Bone resorption was significantly impeded by 50 µM HU (Fig. 5E, F).

These data suggest, HU does not impact the commitment and early differentiation of monocytes into osteoclasts, but rather downregulates late differentiation and bone resorption.

To account for the diminished osteoclast numbers observed by histomorphometry, we further investigated the production of the key osteoclast effector cytokine RANKL by BMSC at 7 days of treatment. HU significantly diminished RANKL production (Fig. 5G).

The data suggest that HU blunts bone resorption by directly inhibiting late differentiation/resorption, and osteoclastogenesis indirectly by inhibiting expression of RANKL by cells of the osteoblast lineage.

### HU impedes BMSC and osteoclast mitochondrial, but not glycolytic energy metabolism

**Fig. 6:**
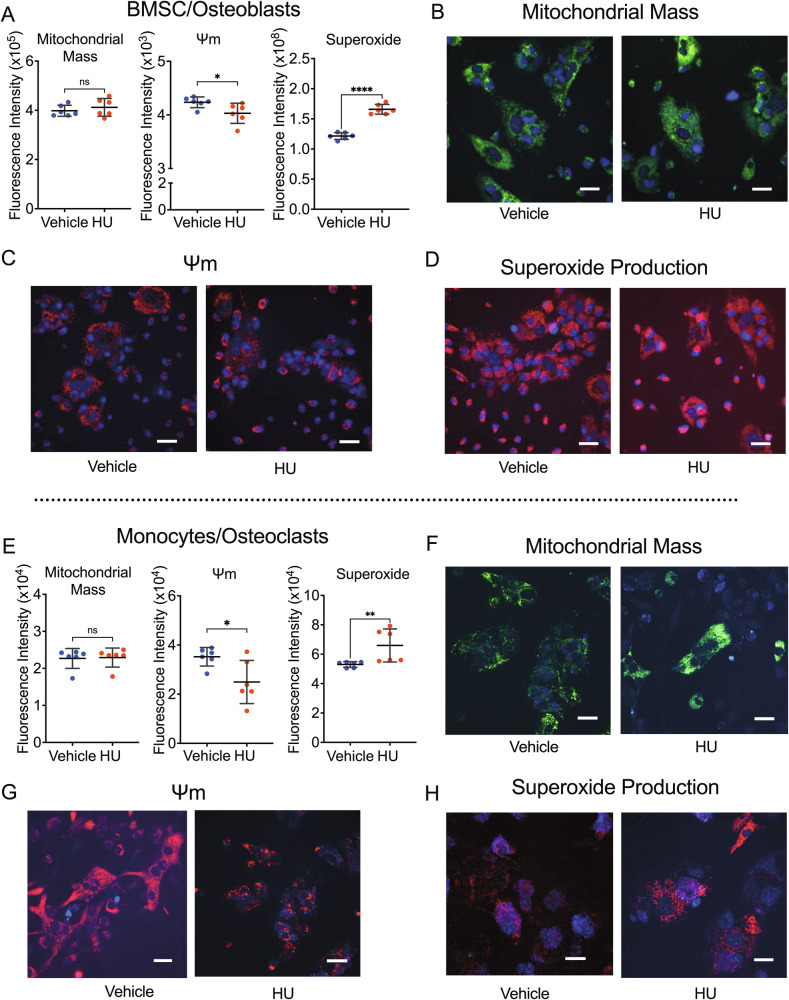
Effect of HU on mitochondrial mass, membrane potential, and superoxide production in BMSCs and osteoclasts. Mitochondrial mass and function in BMSC/osteoblasts (**A**–**D**) and RANKL/M-CSF-treated monocytes**/**osteoclasts (**E**–**H**) exposed to HU (50 µM) for 7 days were quantified by flow cytometry (**A** and **E**) using the fluorescent marker dyes for total mitochondrial mass, mitochondrial membrane potential (Ψm) and mitochondrial-specific superoxide production. Data expressed as Mean ± S.D. *n* = 6 samples/group. Data representative of 3 independent experiments. *p < 0.05, **p < 0.01, ****p < 0.0001. Statistical analyses involved Student’s *t*-test. All data was normally distributed based on the Shapiro–Wilk normality test. Images (60× magnification) were captured using confocal microscopy for Mitochondrial mass (**B** and **F**), Ψm (**C** and **G**) and Superoxide production (**D** and **H**). White scale bars represent 20 µm. Minor contrast and brightness adjustments were performed in PowerPoint to optimize visualization with comparative images adjusted equally.

XTT assays are surrogates for cell number, viability, cytotoxicity, and proliferation. Because XTT directly measures mitochondrial NADH activity (mitochondrial metabolism) [26], we examined whether HU impacts mitochondrial activity. In BMSC and differentiated osteoclasts mitochondrial mass, membrane energy potential (Ψm), and superoxide production were quantified at 7 days after HU treatment (50 µM) by flow cytometry using specific fluorescent dyes (Fig. 6A, E) and visual representations obtained by confocal microscopy (Fig. 6B–D (BMSC) and 6F-H (Osteoclasts)). No significant changes in mitochondrial mass were detected, however, a significant reduction in Ψm and a increase in superoxide production was observed.

**Fig. 7:**
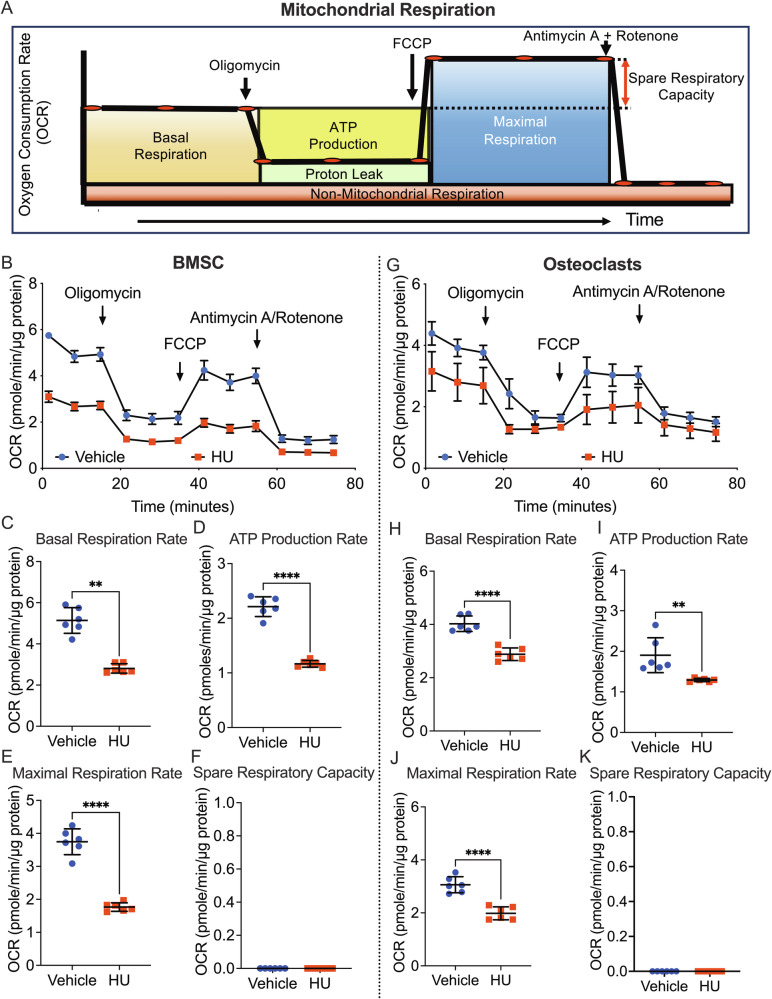
Effect of HU on mitochondrial energy production in BMSCs and osteoclasts. Mitochondrial oxidative phosphorylation in BMSC/osteoblasts (**B**–**F**) and osteoclasts (**G**–**K**) treated with HU (50 µM) for 7 days, was quantified using a Seahorse-XF real-time extracellular flux analyzer. **A** Schematic overview of mitochondrial respiration and quantification of indices, using specific inhibitors of different components of the electron transport chain (oligomycin, FCCP, antimycin A, and rotenone) adapted from Seahorse XF protocol guide (Agilent Technologies, Inc). **B**, **G** Oxygen consumption rate (OCR) profiles over time. Data are expressed as Mean ± SD. *n* = 3 samples/group. **C**, **H** Basal respiration rate, **D**, **I** ATP production rate, **E**, **J** maximal respiration rate, and **F**, **K** spare respiratory capacity. Data are expressed as Mean ± SD. *n* = 6 samples/group combined from 2 independent experiments of *n* = 3 each. **p < 0.01, ***p < 0.01, ****p < 0.0001. Statistical analyses involved Student’s *t*-test or Mann–Whitney test if nonparametric (**I**, **J**) as assessed by the Shapiro–Wilk normality test.

As Ψm is a key determinant of mitochondrial metabolism, we quantified mitochondrial respiration and glycolysis in BMSC and differentiated osteoclasts after 7 days of exposure to 50 µM HU using a Seahorse real-time metabolic analyzer. For mitochondrial assessments we quantified OCR at baseline (basal respiration rate), ATP production rate, maximum respiration rate, and spare respiratory capacity, using the inhibitors oligomycin, FCCP, and antimycin A/rotenone, to interrogate specific points in the electron transport chain (Fig. 7A). Real-time traces of OCR for vehicle and HU-treated BMSC and osteoclasts are shown in Fig. 7B and G respectively. HU significantly blunted basal respiration rate, ATP production rate, and maximum respiration rate, in BMSC (Fig. 7C–E) and in osteoclasts (Fig. 7H–J). No spare respiratory capacity was identified in either cell type (Fig. 7F, K) suggesting energy production was running at maximum.

No effects of HU on glycolysis (ECAR, basal respiration, glycolysis, glycolytic capacity, and glycolytic reserve) were observed in either BMSC or osteoclasts (Supplementary Fig. 5A–F).

The data suggest that HU blunts mitochondrial energy metabolism in BMSC by increasing superoxide production, leading to a decline in Ψm.

## Discussion

This study examined how HU affects the axial skeleton using the Townes SCD mouse model and examined the effects and mechanisms of action of HU on the skeleton using WT mice. Our study focused on HU rather than the actions of SCD, as previous research has already extensively investigated direct SCD-related bone deterioration mechanisms in Townes mice [17, 18, 20, 23]. Our findings reveal that while HU may improve cortical defects caused by SCD in mature mice, HU initiates a low bone turnover state with depressed osteoclast and osteoblast function that leads to decreased trabecular bone mass and compromised microarchitecture.

The beneficial properties of HU on cortical bone are likely mediated through the alleviation of SCD itself. By contrast, HU deteriorates trabecular bone mass by inhibiting osteoblast precursor proliferation and early commitment/differentiation into the osteoblast lineage. Osteoclastogenesis was also impacted, but through both indirect mechanisms, involving diminished RANKL production by osteoblast-lineage cell reducing osteoclast differentiation, and by direct inhibitory effects of HU on activity (resorption) of mature differentiated osteoclasts.

Although our studies demonstrate direct effects of HU on early osteoblast differentiation and osteoclast resorptive activity, bone modeling/remodeling within the basic multicellular unit involves a coordinated response between osteoclasts and osteoblasts together with cross-talk between numerous other cells including osteocytes, osteomacs, vascular endothelial cells and osteocytes [27]. For example, our data reveal that HU can diminish production of RANKL by osteoblast lineage cells further downregulating osteoclastogenesis and resorptive activity. It is further reported that matrix-embedded hypertrophic chondrocytes and osteocytes are essential sources of RANKL [28]. The actions of HU on these other cells that regulate bone homeostasis remain to be studied.

The suppressive activities of HU on both osteoclasts and osteoblasts are likely mediated through the upregulation of superoxide production in the mitochondria, leading to declines in Ψm and mitochondrial energy production. Protein synthesis is among the most energy-costly cellular processes [29, 30] and synthesis of extracellular matrix by osteoblasts necessitates a high mitochondrial Ψm to sustain ATP production [30]. Likewise, osteoclasts also have a high energy demand and mitochondrial respiration and glycolysis play critical roles at different stages of osteoclast maturation [31]. Diminished osteoblast and osteoclast activity following HU treatment, is thus consistent with a decline in energy metabolism.

Although HU revealed evidence of necrotic and/or apoptotic effects at doses of 100 µM and above, the actions of HU on bone cell parameters observed at lower doses (≤50 µM), including energy metabolism, are likely not due to overt toxicity. Our murine BMSC data are further consistent with a study using human BMSC where HU mediated cytostatic rather than cytotoxic actions were reported and led to diminished osteogenic and adipogenic differentiation [32].

Our findings raise concerns regarding chronic HU therapy in children, potentially hindering bone modeling and peak trabecular BMD attainment that is crucial for fracture prevention in adulthood. Moreover, HU adversely affected bone remodeling in mature adult mice, suggesting potential degradation of trabecular bone may occur in adults with SCD on HU therapy. Although we did not observe the inhibitory effects of HU on the cortical compartment, extended use of HU may lead to cortical bone degradation as this compartment is less metabolically active and remodels slower than trabecular bone. Longer-term studies and analysis of bone fracture surrogates are necessary to assess the long-term effects of HU on cortical strength.

Despite significant reductions in bone volume and microarchitecture observed in our mouse study via µCT and histomorphometry, DEXA failed to detect BMD changes, even in trabecular-rich sites like the lumbar spine. A similar outcome was obtained in a clinical study of SCD where BMD analysis by DEXA failed to link HU with low BMD [6]. Despite routine clinical use of DEXA for BMD estimation, it has severe limitations including averaging of cortical and trabecular compartments. Furthermore, in hemoglobinopathies including SCD, hepatic iron overload can cause overestimation of vertebral BMD [33]. Clinical studies using robust tools to assess cortical and trabecular bone compartments, such as quantitative computed tomography, are needed to study bone effects of HU.

In SCD mice, a dose of 40 mg/kg/day (2 times/week) of HU attenuated bone volume, whereas doses of 75–150 mg/kg/day (5 times/week) were required in WT mice. This difference may be attributed to the longer treatment duration of SCD mice (3 months compared to 1 month for WT mice) and/or the pre-existing stress on bone turnover due to SCD, which might lower the threshold for bone loss. Additionally, considering that the SCD mice were males, and the WT mice were females, sex differences which are already reported to affect bone structure in SCD mice [18] may also account for the different bone sensitivities to HU.

Our data showed a reduction in bone turnover by HU, and this is in line with decreased serum markers of resorption reported in a clinical study of HU and bone turnover in people with SCD [34].

In conclusion, HU blunts trabecular bone apposition in the context of SCD and in WT mice, by driving a low bone turnover state. HU likely impedes bone formation and bone resorption, by suppressing mitochondrial energy production. Our findings suggest that HU may impede bone formation in growing children and hinder bone regeneration and fracture repair in adults. If our mouse studies are validated in humans, interventions such as anabolic therapies may be warranted to improve bone health in individuals undergoing HU treatment. Further research in this area will be crucial to guide clinical practice and optimize skeletal outcomes in patients receiving HU therapy.

## Materials and methods

Expanded methodology is provided in a Supplementary Methods section.

### Mice

Mice were housed under specific pathogen-free conditions and were fed gamma-irradiated 5V02 mouse chow (Purina Mills, St. Louis, MO) and autoclaved water ad libitum. The animal facility was kept at 23 °C with 50% relative humidity and a 12/12 h light/dark cycle.

Female C57BL6/J WT and Male “Townes” sickle mice (B6;129-Hbb^tm2(HBG1,HBB*)Tow^/ Hbb^tm3(HBG1,HBB)Tow^ Hba^tm1(HBA)Tow^/J) which develop SCD (“SS” mice) and matched healthy non-sickling control mice (“AA” mice) [16] were from Jackson Laboratory (Bar Harbor, ME). SS or WT C57BL6 mice were administered HU (Sigma-Millipore, St. Louis, MO) dissolved in phosphate-buffered saline (PBS) by IP injection while control groups received only vehicle (PBS). Skeletally immature SS mice (9–11 weeks of age) and SS mice at the onset of skeletal maturity (15–18 weeks of age) received an HU dose of 40 mg/kg/day for 3 months. For mechanistic studies, we used young (8 weeks of age) female WT C57BL6 mice, treated with an HU dose-response comprising 35, 75, or 150 mg/kg/day 5 times/week for 30 days. These doses represent a direct unadjusted weight-based scaling of HU from human to mouse dose (35 mg/kg/day), an allometrically ¾ power scaled dose (75 mg/kg/day) to compensate for the increased metabolic rate of the mouse compared to humans [25] and 150 mg/kg/day, a supra-pharmacological dose to maximize bone effects for optimal sensitivity in mechanistic investigations.

To minimize bias, animals were assigned randomly to experimental groups. µCT and histomorphometry samples were analyzed by an operator blinded to the nature of the samples.

### Micro-computed tomography (µCT)

µCT was performed using a µCT40 scanner (SCANCO Medical, Bassersdorf, Switzerland) as previously described [35, 36].

### Dual-energy X-ray absorptiometry (DEXA)

In vivo BMD of the total body, lumbar spine, and femurs (left and right averaged for each mouse) were made by DEXA using a PIXImus2 bone densitometer (GE Medical Systems, Piscataway, NJ) as described [35, 37].

### Quantitative bone histomorphometry

Bone histomorphometry was performed at the University of Alabama at Birmingham, Center for Metabolic Bone Disease-Histomorphometry and Molecular Analysis Core Laboratory, as previously described [35] using Bioquant-Osteo, (Nashville TN) image analysis software.

### Osteoblast differentiation and mineralization assays

WT BMSC were purified as described [38] and seeded in 24-well plates at 10,000 cells/well for differentiation assays or 25,000 cells/well for mineralization assays in differentiation medium (DM) comprising α-MEM supplemented with 10% FBS and 50 µM ascorbate and 10 mM β-glycerophosphate. Cells were stained at 7 days for alkaline phosphatase activity and at 14 days for mineral deposition, using the 1-Step alkaline phosphatase kit and Alizarin red S, respectively, from Thermo-Fisher Scientific (Waltham, MA). Plates were scanned on a flatbed scanner, and densitometry was performed using ImageJ (V. 1.53 T).

### Real-time RT-PCR

Real-time RT-PCR was performed as described [39, 40] using total RNA extracted with GeneJET RNA purification kit (Thermo-Fisher Scientific). cDNA was synthesized using random hexamers and SuperScript III First-Strand Synthesis System (Thermo-Fisher Scientific) on an ABI Prism-7000 instrument (Applied Biosystems, Foster City, CA) using SYBR green Master Mix (Applied Biosystems) using published primers [41]. Changes were calculated using the 2^−^^ΔΔCT^ method with normalization to 18S rRNA.

### Cellular bioenergetics assays

Real-time ATP production rates were calculated from oxygen consumption rate (OCR) for mitochondrial respiration, and extracellular acidification rate (ECAR) for glycolysis, using a Seahorse XFp extracellular flux analyzer and Wave-Desktop-2.6 software (Agilent Technologies, Santa Clara, CA) with normalization for total protein using a BCA Protein Assay Kit (Thermo-Fisher Scientific).

### Mitochondrial staining

BMSC and osteoclast mitochondrial mass were quantified using MitoTracker-Green-FM (Cell Signaling Technology, Danvers, MA), membrane potential (Ψm) using MitoSpy-Red-CMXRos (BioLegend Inc., San Diego, CA) and mitochondrial-specific superoxide using MitoSox-Red (Invitrogen Corporation, Carlsbad, CA) as described [42]. Mean fluorescence intensity (MFI) was quantified using an Aurora Flow Cytometer (Cytek Biosciences, Fremont, CA) with FlowJo-V10.10.0 software (FlowJo, Ashland, OR). Confocal images of live cells were taken with a Nikon CSU-W1 SoRa confocal field scanning microscope (Nikon Instruments Inc. Melville, NY) using a 60x oil-immersion lens.

### Apoptosis assays

Purified BMSC and splenic monocytes were treated with HU at 50 µM and 100 µM for 7 days. Monocytes received 25 ng/ml M-CSF (R&D Systems). Cells were stained with Annexin V and propidium iodide using a commercial apoptosis detection kit (BD Biosciences, San Diego, CA, USA) according to the manufacturer’s instructions and cells analyzed on a Cytek Aurora flow cytometer (Cytek Biosciences) and using FlowJo Software Version10.10.0 (FlowJo).

.

### In vitro osteoclastogenesis assays

Osteoclastogenesis assays were performed using primary splenic mouse monocytes cultured in α-MEM for 7 days with RANKL (30 ng/ml) and/or M-CSF (25 ng/ml) from R&D Systems (Minneapolis, MN) and stained for Tartrate resistant acid phosphatase (TRAP) after 6 days using a leukocyte acid phosphatase kit (Sigma-Millipore, St. Louis, MO). For actin ring formation, osteoclasts were stained for F-actin with Red Fluorescent Phalloidin Conjugate (Catalog #Ab112127) using the kit of Abcam (Waltham, MA) followed by confocal imaging.

### Osteoclast activity assays

Resorptive activity was quantified using a kit from Cosmo Bio USA (Carlsbad, CA), and resorption pits were visualized by toluidine blue staining and photographed by Nikon Eclipse TE2000-S with Q-color3 camera and Q-Capture-Pro-V7 software. Release of resorption products was quantified by fluorography using 485 nm excitation and 535 nm emission wavelength on a SpectraMax iD3 spectrophotometer (Molecular Devices, San Jose, CA).

### XTT and bromodeoxyuridine (BrdU) assays

XTT and BrdU assays were used to quantify cell proliferation/viability/cytotoxicity using Kits from Abcam (Boston, MA). Absorbance was read at 450 nm on a SpectraMax iD3 spectrophotometer (Molecular Devices).

### Statistical analysis

Significance was determined using Prizm-V10.1 for Macintosh (GraphPad Software Inc., La Jolla, CA). Gaussian distribution was assessed by the Shapiro–Wilk test. Normally distributed two-sample comparisons involved unpaired Student’s *t*-test or Mann–Whitney test for nonparametric data. Multigroup comparisons (≥3 groups) were performed using one-way analysis of variance (ANOVA) with Tukey–Kramer post-test, or Kruskal–Wallis test with Dunn’s post-test if nonparametric. All tests were two-sided, and data presented as mean with error bars representing S.D. *p* < 0.05 was considered statistically significant, and all available data are shown in graphs and used for analysis.

### Estimation of sample size for mice studies

Given the effects of HU on bone mass in SCD and WT mice are unknown it was not possible to predict standard deviation or effect size to perform power analysis. We thus relied on published data involving previous µCT studies of Townes mice where 4–6 Townes mice/group provided statistically significant outcomes for SCD [18]. For WT mice, which are readily available in larger numbers, we used 9–10 mice/group to ensure adequate power to detect small changes in response due to HU administration. The number of animals used for each group in each experiment is stated in the figure legend.

## Supplementary information

Supplemental Figures Revised

Supplemental Expanded Methods Revised

## Data Availability

The datasets generated and/or analyzed during the current study are available from the corresponding author upon reasonable request.

## References

[1] Kavanagh PL, Fasipe TA, Wun T. Sickle cell disease: a review. JAMA. 2022;328:57–68. 35788790 10.1001/jama.2022.10233

[2] Lanzkron S, Strouse JJ, Wilson R, Beach MC, Haywood C, Park H, et al. Systematic review: Hydroxyurea for the treatment of adults with sickle cell disease. Ann Intern Med. 2008;148:939–55. 18458272 10.7326/0003-4819-148-12-200806170-00221 PMC3256736

[3] Green NS, Barral S. Emerging science of hydroxyurea therapy for pediatric sickle cell disease. Pediatr Res. 2014;75:196–204. 24252885 10.1038/pr.2013.227 PMC3917141

[4] Miller RG, Segal JB, Ashar BH, Leung S, Ahmed S, Siddique S, et al. High prevalence and correlates of low bone mineral density in young adults with sickle cell disease. Am J Hematol. 2006;81:236–41. 16550513 10.1002/ajh.20541

[5] Sadat-Ali M, Al-Elq A, Sultan O, Al-Turki H. Secondary osteoporosis due to sickle cell anemia: do sex steroids play a role? Indian J Med Sci. 2008;62:193–8. 18579978

[6] Sarrai M, Duroseau H, D’Augustine J, Moktan S, Bellevue R. Bone mass density in adults with sickle cell disease. Br J Haematol. 2007;136:666–72. 17223909 10.1111/j.1365-2141.2006.06487.x

[7] Meeuwes M, Souza de Carvalho TF, Cipolotti R, Gurgel RQ, Ferrao TO, Peters M, et al. Bone mineral density, growth, pubertal development and other parameters in Brazilian children and young adults with sickle cell anaemia. Trop Med Int Health. 2013;18:1539–46. 24134458 10.1111/tmi.12211

[8] Voskaridou E, Stoupa E, Antoniadou L, Premetis E, Konstantopoulos K, Papassotiriou I, et al. Osteoporosis and osteosclerosis in sickle cell/beta-thalassemia: the role of the RANKL/osteoprotegerin axis. Haematologica. 2006;91:813–6. 16704959

[9] Adewoye AH, Chen TC, Ma Q, McMahon L, Mathieu J, Malabanan A, et al. Sickle cell bone disease: response to vitamin D and calcium. Am J Hematol. 2008;83:271–4. 17924548 10.1002/ajh.21085

[10] Lal A, Fung EB, Pakbaz Z, Hackney-Stephens E, Vichinsky EP. Bone mineral density in children with sickle cell anemia. Pediatr Blood Cancer. 2006;47:901–6. 16317761 10.1002/pbc.20681

[11] Chapelon E, Garabedian M, Brousse V, Souberbielle JC, Bresson JL, de Montalembert M. Osteopenia and vitamin D deficiency in children with sickle cell disease. Eur J Haematol. 2009;83:572–8. 19682065 10.1111/j.1600-0609.2009.01333.x

[12] Kooy A, de Heide LJ, ten Tije AJ, Mulder AH, Tanghe HL, Kluytmans JA, et al. Vertebral bone destruction in sickle cell disease: infection, infarction or both. Neth J Med. 1996;48:227–31. 8710044 10.1016/0300-2977(95)00075-5

[13] Osunkwo I, Hodgman EI, Cherry K, Dampier C, Eckman J, Ziegler TR, et al. Vitamin D deficiency and chronic pain in sickle cell disease. Br J Haematol. 2011;153:538–40. 21275953 10.1111/j.1365-2141.2010.08458.x

[14] Fung EB, Harmatz PR, Milet M, Coates TD, Thompson AA, Ranalli M, et al. Fracture prevalence and relationship to endocrinopathy in iron overloaded patients with sickle cell disease and thalassemia. Bone. 2008;43:162–8. 18430624 10.1016/j.bone.2008.03.003 PMC2500183

[15] Almeida A, Roberts I. Bone involvement in sickle cell disease. Br J Haematol. 2005;129:482–90. 15877730 10.1111/j.1365-2141.2005.05476.x

[16] Wu LC, Sun CW, Ryan TM, Pawlik KM, Ren J, Townes TM. Correction of sickle cell disease by homologous recombination in embryonic stem cells. Blood. 2006;108:1183–8. 16638928 10.1182/blood-2006-02-004812 PMC1895869

[17] Xiao L, Zhou Y, Bokoliya S, Lin Q, Hurley M. Bone loss is ameliorated by fecal microbiota transplantation through SCFA/GPR41/ IGF1 pathway in sickle cell disease mice. Sci Rep. 2022;12:20638. 36450880 10.1038/s41598-022-25244-9 PMC9712597

[18] Selma J, Song H, Rivera C, Douglas S, Akella A, Bollavaram K, et al. Sickle cell disease promotes sex-dependent pathological bone loss through enhanced cathepsin proteolytic activity in mice. Blood Adv. 2022;6:1381–93. 34547771 10.1182/bloodadvances.2021004615 PMC8905708

[19] Rana K, Pantoja K, Xiao L. Bone marrow neutrophil aging in sickle cell disease mice is associated with impaired osteoblast functions. Biochem Biophys Rep. 2018;16:110–4. 30417128 10.1016/j.bbrep.2018.10.009 PMC6214830

[20] Xiao L, Andemariam B, Taxel P, Adams DJ, Zempsky WT, Dorcelus V, et al. Loss of bone in sickle cell trait and sickle cell disease female mice is associated with reduced IGF-1 in bone and serum. Endocrinology. 2016;157:3036–46. 27171384 10.1210/en.2015-2001

[21] Lopes FC, Traina F, Almeida CB, Leonardo FC, Franco-Penteado CF, Garrido VT, et al. Key endothelial cell angiogenic mechanisms are stimulated by the circulating milieu in sickle cell disease and attenuated by hydroxyurea. Haematologica. 2015;100:730–9. 25769545 10.3324/haematol.2014.119727 PMC4450618

[22] Green M, Akinsami I, Lin A, Banton S, Ghosh S, Chen B, et al. Microarchitectural and mechanical characterization of the sickle bone. J Mech Behav Biomed Mater. 2015;48:220–8. 25957113 10.1016/j.jmbbm.2015.04.019 PMC4442736

[23] Dalle Carbonare L, Matte A, Valenti MT, Siciliano A, Mori A, Schweiger V, et al. Hypoxia-reperfusion affects osteogenic lineage and promotes sickle cell bone disease. Blood. 2015;126:2320–8. 26330244 10.1182/blood-2015-04-641969

[24] Maupin KA, Childress P, Brinker A, Khan F, Abeysekera I, Aguilar IN, et al. Skeletal adaptations in young male mice after 4 weeks aboard the International Space Station. NPJ Microgravity. 2019;5:21. 31583271 10.1038/s41526-019-0081-4 PMC6760218

[25] West GB, Brown JH. The origin of allometric scaling laws in biology from genomes to ecosystems: towards a quantitative unifying theory of biological structure and organization. J Exp Biol. 2005;208:1575–92. 15855389 10.1242/jeb.01589

[26] Berridge MV, Herst PM, Tan AS. Tetrazolium dyes as tools in cell biology: new insights into their cellular reduction. Biotechnol Annu Rev. 2005;11:127–52. 16216776 10.1016/S1387-2656(05)11004-7

[27] Kular J, Tickner J, Chim SM, Xu J. An overview of the regulation of bone remodelling at the cellular level. Clin Biochem. 2012;45:863–73. 22465238 10.1016/j.clinbiochem.2012.03.021

[28] Xiong J, Onal M, Jilka RL, Weinstein RS, Manolagas SC, O’Brien CA. Matrix-embedded cells control osteoclast formation. Nat Med. 2011;17:1235–41. 21909103 10.1038/nm.2448 PMC3192296

[29] Buttgereit F, Brand MD. A hierarchy of ATP-consuming processes in mammalian cells. Biochem J. 1995;312:163–7. 7492307 10.1042/bj3120163 PMC1136240

[30] Dirckx N, Moorer MC, Clemens TL, Riddle RC. The role of osteoblasts in energy homeostasis. Nat Rev Endocrinol. 2019;15:651–65. 31462768 10.1038/s41574-019-0246-y PMC6958555

[31] Estell E, Ichikawa T, Giffault P, Bonewald L, Spiegelman B, Rosen C. Irisin enhances mitochondrial function in osteoclast progenitors during differentiation. Biomedicines. 2023;11:3311. 10.3390/biomedicines11123311 PMC10741766 38137532

[32] Kapor S, Vukotic M, Suboticki T, Dikic D, Mitrovic Ajtic O, Radojkovic M, et al. Hydroxyurea induces bone marrow mesenchymal stromal cells senescence and modifies cell functionality in vitro. J Pers Med. 2021;11:1048. 10.3390/jpm11111048 PMC8619969 34834400

[33] Allard HM, Calvelli L, Weyhmiller MG, Gildengorin G, Fung EB. Vertebral bone density measurements by DXA are influenced by hepatic iron overload in patients with hemoglobinopathies. J Clin Densitom. 2019;22:329–37. 10.1016/j.jocd.2018.07.001 PMC6447456 30122533

[34] Mokhtar GM, Tantawy AA, Hamed AA, Adly AA, Ismail EA, Makkeyah SM. Tartrate-resistant acid phosphatase 5b in young patients with sickle cell disease and trait siblings: relation to vasculopathy and bone mineral density. Clin Appl Thromb Hemost. 2017;23:64–71. 26149452 10.1177/1076029615594001

[35] Ofotokun I, Titanji K, Vikulina T, Roser-Page S, Yamaguchi M, Zayzafoon M, et al. Role of T-cell reconstitution in HIV-1 antiretroviral therapy-induced bone loss. Nat Commun. 2015;6:8282. 26392000 10.1038/ncomms9282 PMC4580984

[36] Roser-Page S, Vikulina T, Zayzafoon M, Weitzmann MN. CTLA-4Ig-induced T cell anergy promotes Wnt-10b production and bone formation in a mouse model. Arthritis Rheumatol. 2014;66:990–9. 24757150 10.1002/art.38319 PMC3994890

[37] Li Y, Toraldo G, Li A, Yang X, Zhang H, Qian WP, et al. B cells and T cells are critical for the preservation of bone homeostasis and attainment of peak bone mass in vivo. Blood. 2007;109:3839–48. 17202317 10.1182/blood-2006-07-037994 PMC1874582

[38] Gao Y, Wu X, Terauchi M, Li JY, Grassi F, Galley S, et al. T cells potentiate PTH-induced cortical bone loss through CD40L signaling. Cell Metab. 2008;8:132–45. 18680714 10.1016/j.cmet.2008.07.001 PMC2569843

[39] Roser-Page S, Weiss D, Vikulina T, Yu M, Pacifici R, Weitzmann MN. Cyclic adenosine monophosphate (cAMP)-dependent phosphodiesterase inhibition promotes bone anabolism through CD8^+^ T cell Wnt-10b production in mice. JBMR. 2022;6:e10636. 10.1002/jbm4.10636 PMC9289889 35866149

[40] Weitzmann MN, Roser-Page S, Vikulina T, Weiss D, Hao L, Baldwin WH, et al. Reduced bone formation in males and increased bone resorption in females drive bone loss in hemophilia A mice. Blood Adv. 2019;3:288–300. 30700417 10.1182/bloodadvances.2018027557 PMC6373738

[41] Bedi B, Li JY, Tawfeek H, Baek KH, Adams J, Vangara SS, et al. Silencing of parathyroid hormone (PTH) receptor 1 in T cells blunts the bone anabolic activity of PTH. Proc Natl Acad Sci USA. 2012;109:E725–33. 22393015 10.1073/pnas.1120735109 PMC3311391

[42] Monteiro LB, Davanzo GG, de Aguiar CF, Moraes-Vieira PMM. Using flow cytometry for mitochondrial assays. MethodsX. 2020;7:100938. 32551241 10.1016/j.mex.2020.100938 PMC7289760

