# Supplementary material for: Hydroxyurea blunts mitochondrial energy metabolism and osteoblast and osteoclast differentiation exacerbating trabecular bone loss in sickle cell mice
Source: Cell Death Dis. 2024 Dec 18;15(12):907. doi: 10.1038/s41419-024-07296-z (PMC11655664; doi:10.1038/s41419-024-07296-z)
Supplement: Supplementary file 1 — Supplemental Figures Revised [file 41419_2024_7296_MOESM1_ESM.pdf]

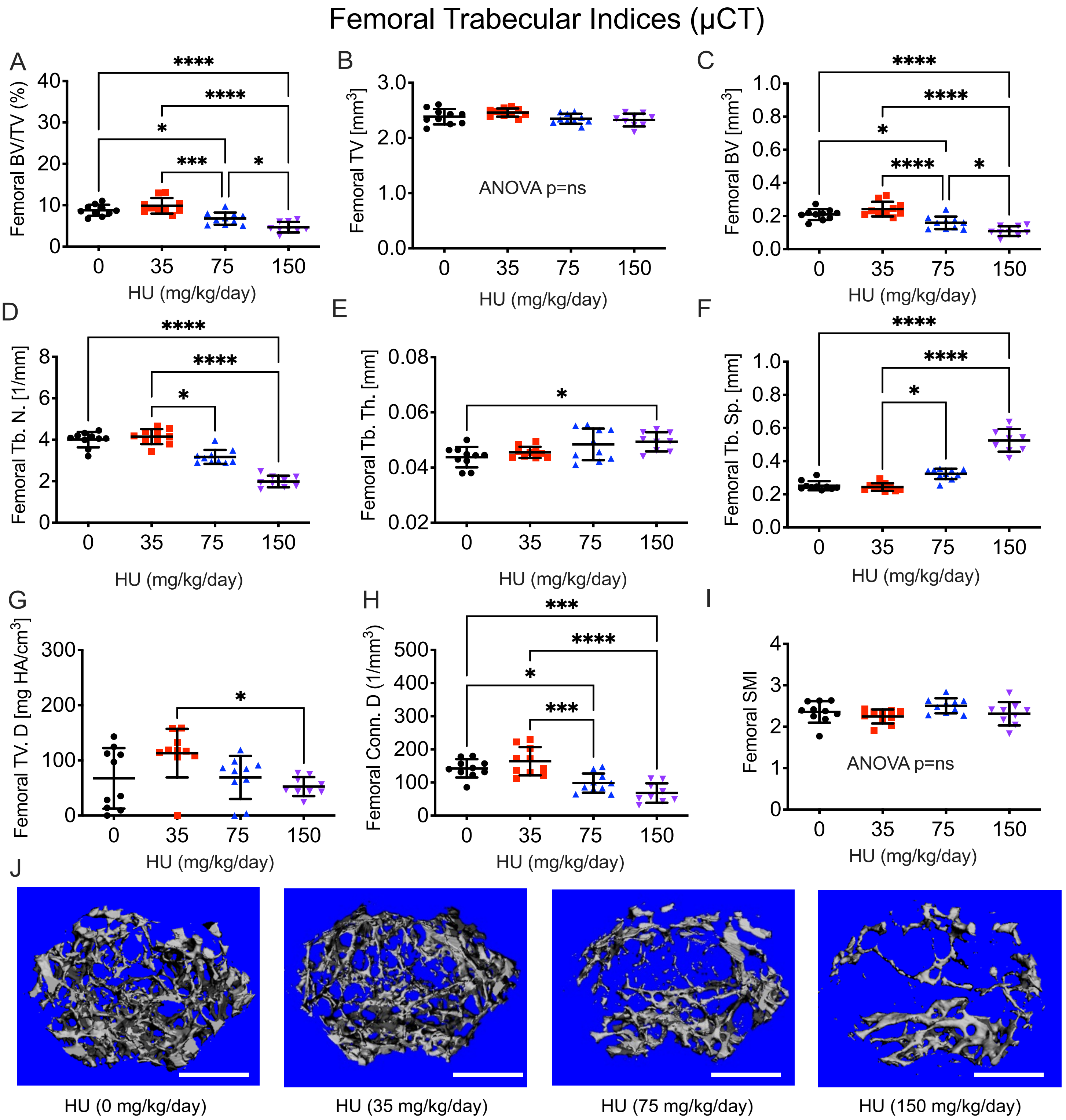

Supplementary Figure 1: Ex vivo μCT analysis of trabecular bone from WT mice femurs treated with HU. Femurs from WT female C57BL6/J mice (8 weeks of age) treated for 30 days with HU (35, 75 and 150mg/Kg/week) and analyzed by high-resolution (6μm) μCT. (A) BV/TV, (B) TV, (C) BV, (D) Tb.N, (E) Tb.Th, (F) Tb.Sp, (G) TV.D, (H) Conn.D, and (I) SMI. (J) Representative high-resolution (6-μm) 3D visual reconstructions of L3 vertebrae. n=9-10 mice/group. White scale lines represent 500μm. Data expressed as Mean±S.D. *p<0.05, ***p<0.001, ****p<0.0001. Non-significant comparisons are not shown. Data analyzed by one-way ANOVA with Tukey post hoc test or Kruskal-Wallis with Dunn's post hoc test for nonparametric data [(D) Tb.N, (F) Tb.Sp, and (G) TV.D] based on Shapiro-Wilk normality test.

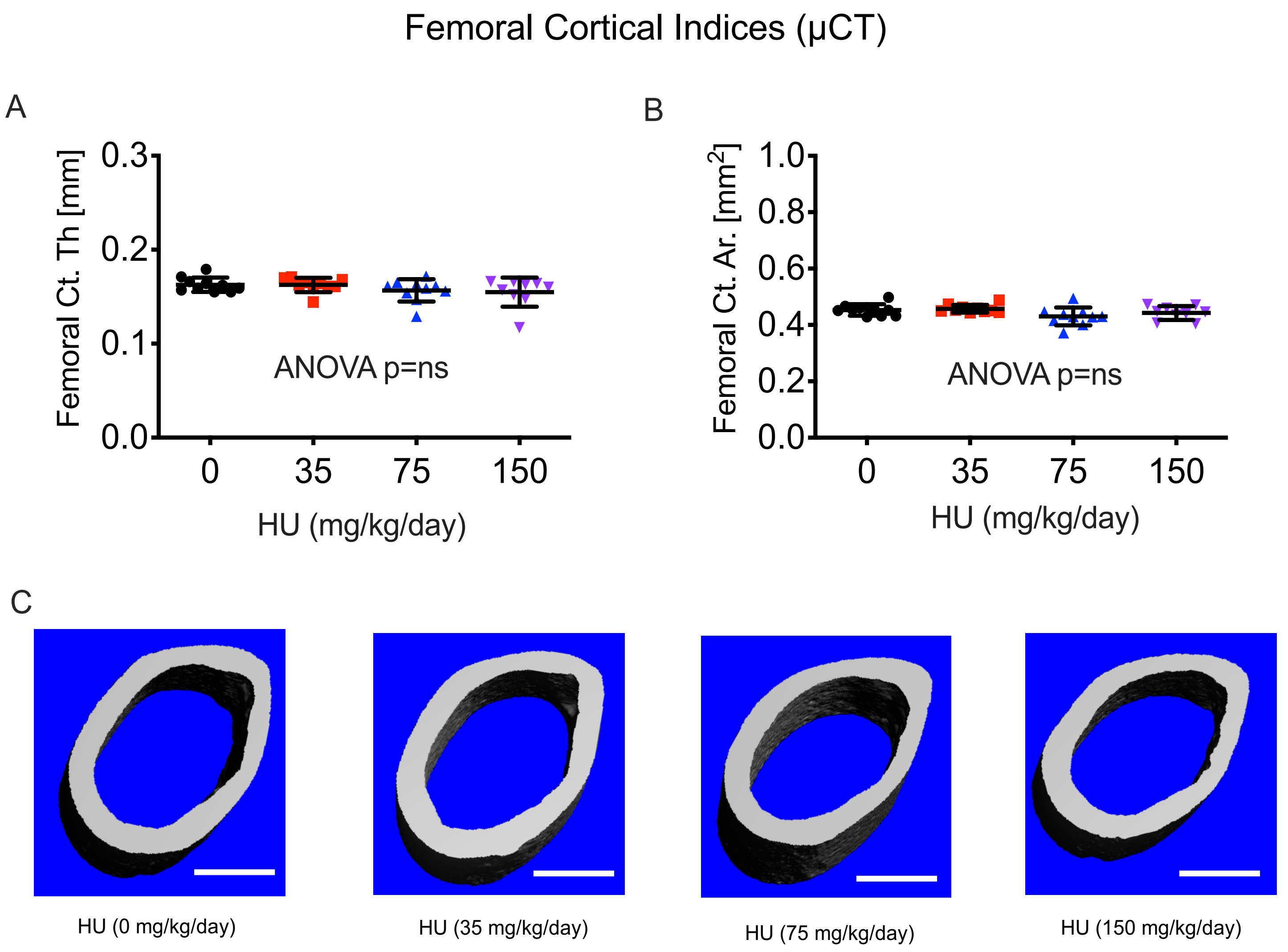

Supplementary Figure 2: Ex vivo µCT analysis of cortical bone from WT mice femurs treated with HU. Femurs from WT female C57BL6/J mice (8 weeks of age) treated for 30 days with HU (35, 75 and 150mg/Kg/week) were analyzed by high-resolution (6µm) µCT at the mid-diaphysis for cortical bone. (A) Ct.Th., (B) Ct.Ar., (C) Representative high-resolution (6-µm) 3D visual reconstructions of Femora cortical bone. n=9-10 mice/group.
White scale lines represent 500µm. Data expressed as Mean±S.D. *$p<0.05$, ***$p<0.001$, ****$p<0.0001$. Non-significant comparisons are not shown. One-way ANOVA with Tukey post hoc test or Kruskal-Wallis with Dunn's post hoc test for nonparametric data [(D) Tb.N, (F) Tb.Sp, and (G) TV.D] based on Shapiro-Wilk normality test.

Bone Mineral Density Quantification by Dual-Energy X-ray Absorptiometry (DEXA)

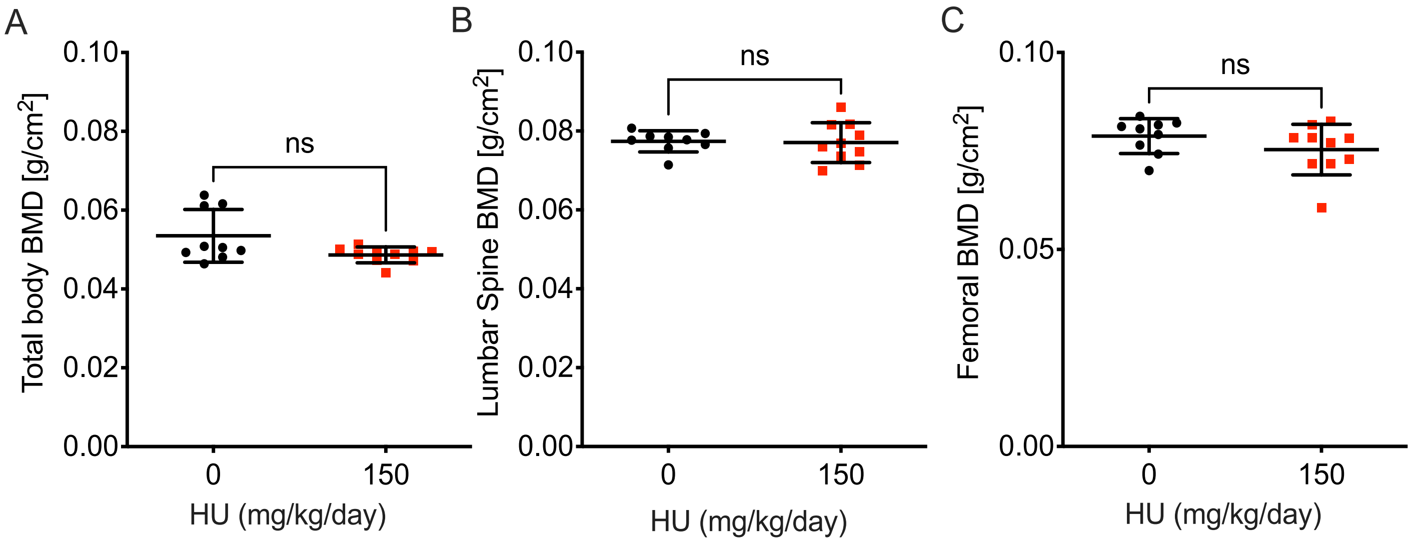

Supplementary Figure 3: Bone Mineral Density Quantification by Dual-Energy X-ray Absorptiometry (DEXA). BMD was quantified for vehicle and mice treated with 150 mg/kg/day after 4 weeks using DEXA. (A) Total body, (B) Lumbar Spine, and (C) Femur (left and right femur averaged for each mouse). N=9-10 mice/group. Mean±SD. p=not-significant (ns). Data analyzed by Student t test. All data analyzed for parametric distribution by Shapiro-Wilk normality test.

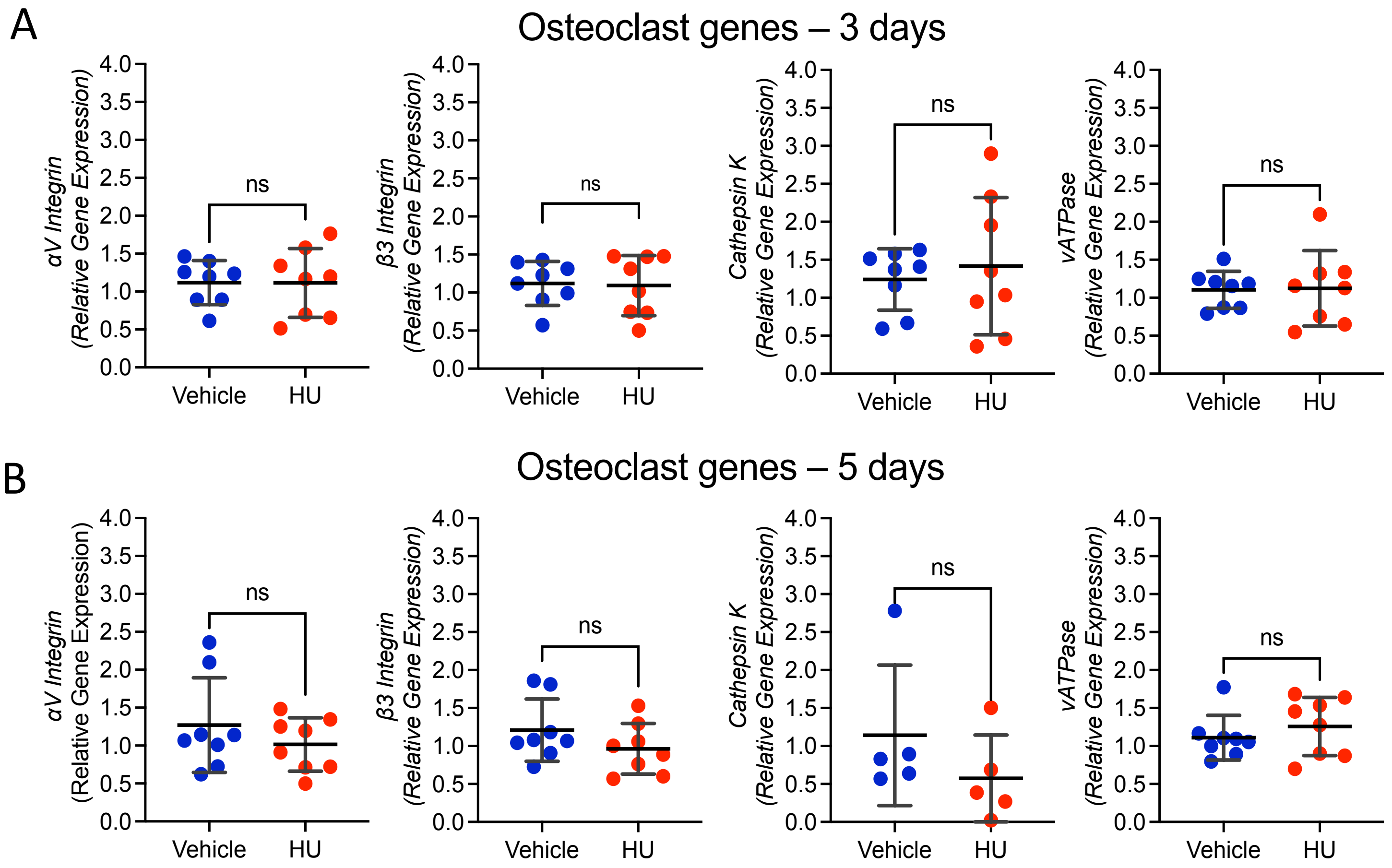

Supplementary Figure 4: Effect of HU on osteoclast gene expression at 3 and 5 days of differentiation. Purified monocytes isolated from WT mouse spleen were cultured with M-CSF and RANKL, and with or without HU (50μM). Osteoclast gene expression was quantified by real-time RT-PCR for αV integrin subunit, β3 integrin subunit, cathepsin K, and vATPase at 3 and 5 days of HU treatment. n=8 data points combined from 2 independent experiments of n=4 each (3 samples from cathepsin K failed to amplify). Data expressed as Mean±S.D. Statistical analyses involved Student's t-test or Mann-Whitney test for nonparametric data (Cathepsin K and vATPase at 3-days). Normal distribution was assessed by the Shapiro-Wilk normality test. p=ns (not significant).

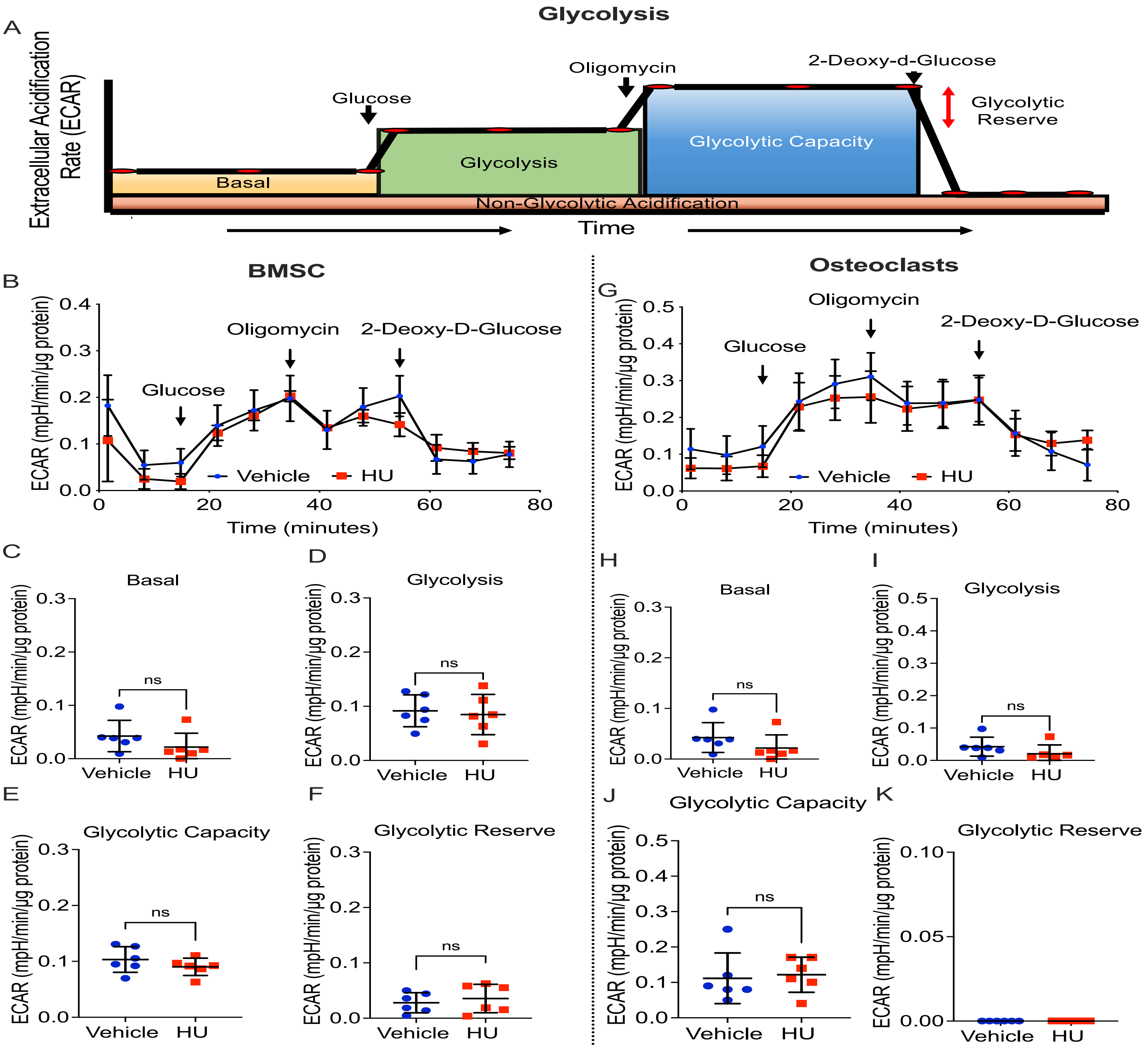

Supplementary Figure 5: Effect of HU on glycolysis in BMSC/Osteoblasts and Osteoclasts. Glycolytic energy metabolism in HU (50 µM) treated BMSC/osteoblasts (B-F) and osteoclasts (G-K) was quantified at 7 days by Seahorse analyzer. (A) Schematic overview of glycolytic respiration and quantification of glycolytic indices, using specific inhibitors of different components of the electron transport chain (oligomycin) or stimulation or inhibition of glycolysis (glucose and 2-deoxy-d-glucose, respectively) Adapted from Seahorse XF protocol guide (Agilent Technologies, Inc). (B and G) extracellular acidification rate (ECAR) profiles over time. n=3 samples/group. (C and H) basal glycolytic rate, (D and I) glycolysis, (E and J) maximal glycolytic capacity, and (F and K) glycolytic reserve. n=6 samples/group combined from 2 independent experiments of n=3 each. Mean±SD. p=not-significant (ns). Data analyzed by Student's t-test based on Shapiro-Wilk normality test. Data representative of at least 2 independent experiments.

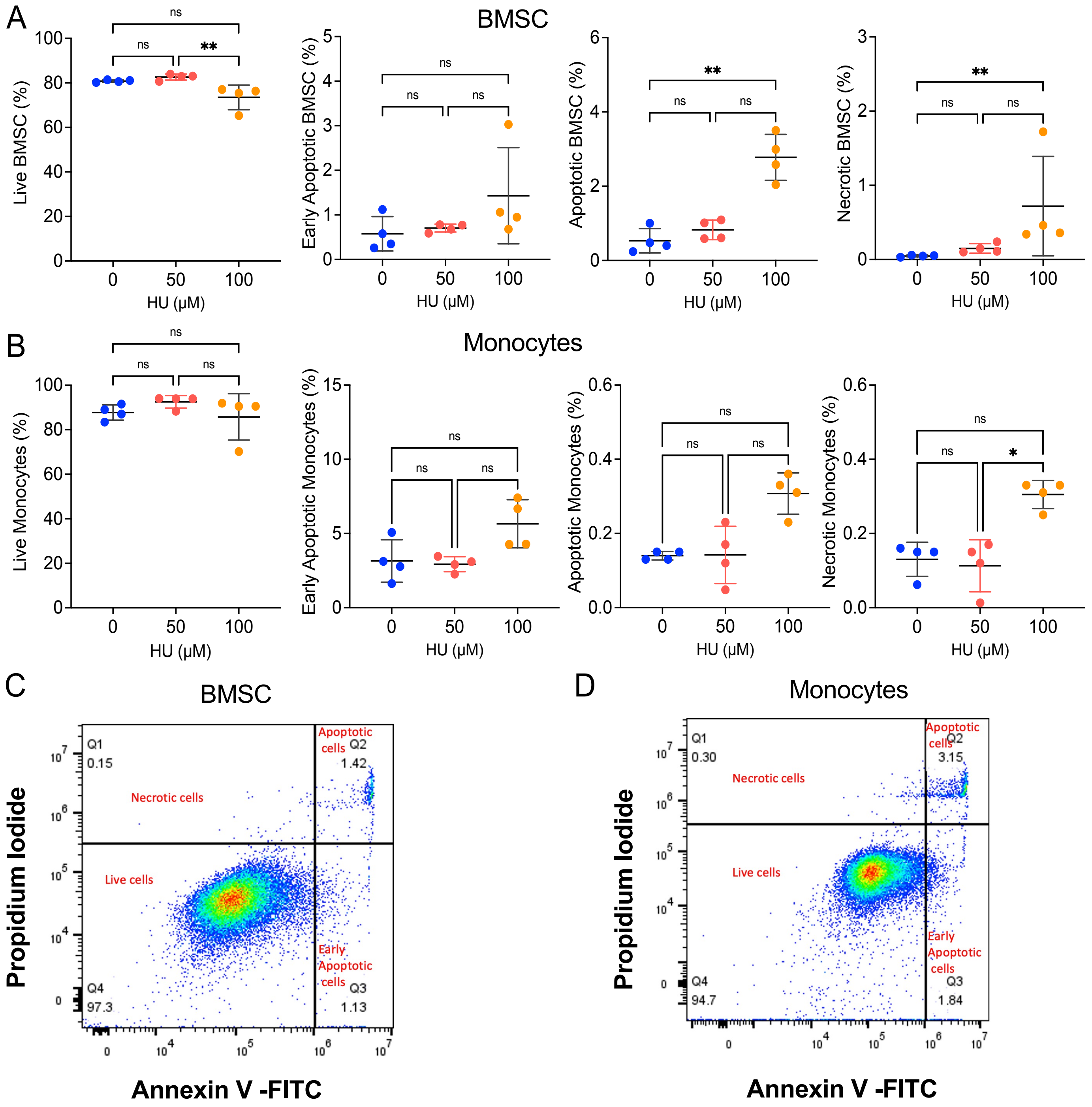

Supplementary Figure 6: Effect of HU on BMSC and Monocyte cell viability. Cell viability in control (0 μM) and HU (50μM and 100μM) treated BMSC (A) and monocytes (B) was quantified following 7 days of HU treatment by Flow Cytometry using Annexin-V and propidium iodide staining. The percentage of live cells, early apoptotic cells, apoptotic cells and necrotic cells was quantified for BMSC and monocytes. n=4 independent biological replicates/group and is representative of 2 independent experiments. Data expressed as Mean±S.D. *$p<0.05$, ***$p<0.001$. p=ns (non-significant). Kruskal-Wallis with Dunn's post hoc test for nonparametric data. Flow gating strategy is shown in representative plots for (C) BMSC and (D) monocytes.
