# Supplementary material for: Hydroxyurea blunts mitochondrial energy metabolism and osteoblast and osteoclast differentiation exacerbating trabecular bone loss in sickle cell mice
Source: Cell Death Dis. 2024 Dec 18;15(12):907. doi: 10.1038/s41419-024-07296-z (PMC11655664; doi:10.1038/s41419-024-07296-z)
Supplement: Supplementary file 2 — Supplemental Expanded Methods Revised [file 41419_2024_7296_MOESM2_ESM.pdf]

## SUPPLEMENTAL METHODS

The animal studies were approved by the Emory University Institutional Animal Care and Use Committee and conducted in accordance with the National Institutes of Health's Laboratory Guide for the Care and Use of Laboratory Animals. Mice were housed under specific pathogen–free conditions and were fed gamma-irradiated 5V02 mouse chow (Purina Mills, St. Louis, MO) and autoclaved water ad libitum. The animal facility was kept at 23°C with 50% relative humidity and a 12/12hr light/dark cycle.

**Mice**: Female C57BL6/J WT and Male "Townes" sickle mice (B6;129-$Hbb^{tm2(HBG1,HBB*)Tow}$/ $Hbb^{tm3(HBG1,HBB)Tow}$ $Hba^{tm1(HBA)Tow}$/J) which develop SCD ("SS" mice) and matched healthy non-sickling control mice ("AA" mice)[1] were from Jackson Laboratory (Bar Harbor, ME). SS or WT C57BL6 mice were administered HU (Sigma-Millipore, St. Louis, MO) dissolved in phosphate-buffered saline (PBS) by IP injection while control groups received only vehicle (PBS). Skeletally immature SS mice (9-11 weeks-of-age) and SS mice at the onset of skeletal maturity (15-18 weeks-of-age) received an HU dose of 40mg/kg/day for 3 months. For mechanistic studies, we used young (8 weeks-of-age) female WT C57BL6 mice, treated with an HU dose-response comprising 35, 75, or 150mg/kg/day 5 times/week for 30 days. These doses represent a direct unadjusted weight-based scaling of HU from human to mouse dose (35mg/kg/day), an allometrically ¾ power scaled dose (75mg/kg/day) to compensate for the increased metabolic rate of the mouse compared to humans[2] and 150mg/kg/day, a supra-pharmacological dose to maximize bone effects for optimal sensitivity in mechanistic investigations.

**Micro-computed tomography (µCT).** µCT was performed using a µCT40 scanner (SCANCO Medical, Bassersdorf, Switzerland) as previously described[3, 4]. Briefly, µCT was performed in vertebrae and femurs ex vivo using a µCT40 scanner (Scanco Medical, Bassersdorf, Switzerland) calibrated weekly with a factory-supplied phantom. For trabecular bone, 200 tomographic slices were taken (total area of 1,200µm) at the distal right femoral metaphysis or the L3 vertebrae, at a voxel size of 6µm (70kVp and 114mA, and 200ms integration time). Trabecular bone was segmented from the cortical shell, for a total area of 1.2 mm beginning ~0.5 mm from the distal growth plate and projection images reconstructed using the auto-contour function for trabecular bone. Cortical bone was quantified at the mid-diaphysis at a voxel size of 6µm. Thresholding was performed by visual inspection of preview and slice-wise gray-scale 2D-images, as recommended by the µCT manufacturer and was maintained consistent for all measurements. Representative samples were reconstructed in 3D to generate visual representations of cortical and trabecular structures.

**Dual-Energy X-ray Absorptiometry (DEXA).** In vivo BMD of total body, lumbar spine, and femurs (left and right averaged for each mouse) were made by DEXA using a PIXImus2 bone densitometer (GE Medical Systems, Piscataway, NJ) as described[3, 5].

**Quantitative bone histomorphometry**. Bone histomorphometry was performed at the University of Alabama at Birmingham, Center for Metabolic Bone Disease-Histomorphometry and Molecular Analysis Core Laboratory, as previously described[3] using Bioquant-Osteo, (Nashville TN) image analysis software.

**Osteoblast differentiation and mineralization assays**. WT bone marrow stromal cells (BMSC) were purified as described[6] and seeded in 24-well plates at 10,000cells/well for

differentiation assays or 25,000cells/well for mineralization assays in differentiation medium (DM) comprising α-MEM supplemented with 10% FBS and 50μM/mL ascorbate and 10mM β-glycerophosphate. Cells were stained at 7 days for alkaline phosphatase activity and at 14-days for mineral deposition, using the 1-Step alkaline phosphatase kit and Alizarin red S, respectively, from Thermo-Fisher Scientific (Waltham, MA). Plates were scanned on a flatbed scanner and densitometry performed using ImageJ (V. 1.53T) to quantify alkaline phosphatase and Alizarin red S staining intensity.

**Real-time RT-PCR.** RT-PCR was performed as described [7, 8]. Total RNA was extracted with GeneJET RNA purification kit (Thermo-Fisher Scientific) and cDNA synthesized using random hexamers and SuperScript III First-Strand Synthesis System (Thermo-Fisher Scientific) on an ABI Prism 7000 instrument (Applied Biosystems, Foster City, CA, USA) using SYBR green Master Mix (Applied Biosystems). Changes in relative gene expression between control and HU-treated groups were calculated using the $2^{-\Delta\Delta CT}$ method with normalization to 18S rRNA. We used the following previously validated primers[9] for osteoblast-related genes: ***Col1a1*** 5′-CCCTACTCAGCCGTCTGTGC-3′ (forward) and 5′-GGGTTCGGGCTGATGTACC-3′ (reverse); ***Osx*** 5′-GTGTTAG- TAACCTGGCCGGG-3′ (forward) and 5′-CATTGGACTTCC-CCCTTCTTG-3′ (reverse); ***Runx2*** 5′-CTGTGGTTACCGTCATGGCC-3′ (forward) and 5′-GGAGCTCGGCGGAGTAGTTC-3′ (reverse). ***Rankl***, 5′-GCACTCCAACTGCCCAAGA-3′ (forward) and 5′-TTTTGGAGCCCTGCTTTCTG-3′ (reverse). ***18S rRNA***: 5′-ATTCGAACGTCTGCCCTATCA-3′ (forward) and 5′-GTCACCCGTGGTCACCATG-3′ (reverse). The following previously published primer sequences were used for osteoclast-related genes: ***Cathepsin K****:* 5'- CCTGCTGTTGAGAATCTGTTCGCTAGGCTC -3 (forward) and 5′ - GTGGCTACTGTGAGCGGAAGACTAAGGGTG -3' (reverse)[10]; ***β3-integrin***: 5′-

CCACTGATGCCAAGACCCATA-3′ (forward) and 5′- TGGCAGCGCCCATCAT-3′ (reverse)[11]; ***αV-integrin***: 5′-CAGTGGCCTTACAAATACAACAACA-3′ (forward) and 5′-TGCAGTTCATTGGTCCATCAA-3′ (reverse)[11]; ***vATPase***: 5′-GTGAGACCTTGGAAGTCCTGAA-3′ (forward) and 5′- GAGAAATGTGCTCAGGGGCT-3′ (reverse)[12].

**Mitochondrial Functional Parameter Quantification**: BMSC and osteoclast mitochondrial mass was quantified using MitoTracker-Green-FM (Cell Signaling Technology, Danvers, MA), membrane potential (Ψm) using MitoSpy-Red-CMXRos (BioLegend Inc., San Diego, CA) and mitochondrial specific superoxide using MitoSox-Red (Invitrogen Corporation, Carlsbad, CA) as described[13]. Mean fluorescence intensity (MFI) was quantified using an Aurora Flow Cytometer (Cytek Biosciences, Fremont, CA) with FlowJo-V10.10.0 software (FlowJo, Ashland, OR).

**Cellular bioenergetics assays.** Real-time ATP production rates were calculated from oxygen consumption rate (OCR) for mitochondrial respiration, and extracellular acidification rate (ECAR) for glycolysis, using a Seahorse XFp extracellular flux analyzer (Agilent Technologies, Santa Clara, CA). BMSC were seeded in Seahorse cell culture miniplates (8 well plates) at 10,000 cells/well in DM and treated with HU (50µM) for 7 days. Immediately before extracellular flux analysis, DM was replaced with Seahorse XF DMEM medium pre-adjusted to pH 7.4 and supplemented with 10mM glucose, 1mM pyruvate and 2mM glutamine. Basal OCR and ECAR measurements were taken and then followed by serial injections of 1.0µM oligomycin (an ATP synthase inhibitor), 1, 2, 4µM carbonyl cyanide p-trifluoro-methoxyphenyl hydrazone (FCCP) a mitochondrial uncoupler and a mixture of 0.5µM rotenone and 0.5µM antimycin A (inhibitors of Complex I and III of the electron transport chain, respectively). Glycolysis was measured by serial

addition of 10mM glucose, 1.0μM oligomycin and 50mM 2-Deoxy-D-glucose (2-DG). All additives were from Agilent Technologies. Mitochondrial, glycolytic, and total ATP production rates were quantified according to the manufacturer's protocol normalizing for total protein content using a BCA Protein Assay Kit (Thermo Fisher Scientific, Waltham, MA, USA) with absorbance read at 562 nm using a SpectraMax microplate reader running SoftMax Pro 5.3 software (Molecular Devices, San Jose, CA, USA). Energy production indices were calculated using Wave Desktop 2.6 (Agilent Technologies, Santa Clara, CA).

**In vitro osteoclastogenesis assays.** Osteoclastogenesis assays were performed using primary splenic mouse monocytes cultured in α-MEM supplemented with 10% FBS for 7-days with RANKL (30ng/ml) and/or M-CSF (25ng/ml) from R&D Systems (Minneapolis, MN) and stained for Tartrate resistant acid phosphatase (TRAP) after 6-days using a leukocyte acid phosphatase kit (Sigma-Millipore, St. Louis, MO).

**Osteoclast F-actin ring immunohistochemistry.** Osteoclasts generated as described above and treated with or without HU (10μM) were subjected to formaldehyde fixation by incubating cells in 4.0% formaldehyde in PBS at room temperature for 10-minutes. After rinsing the fixed cells twice in PBS, cells were permeabilized with 0.1% Triton X-100 for 5 minutes, followed by two rinses with PBS. Finally, the cells were stained with 1x Red Fluorescent Phalloidin Conjugate (Catalog #Ab112127) for 60 minutes using an F-actin staining kit from Abcam (Waltham, MA) before confocal imaging.

**Osteoclast Activity Assays:** Resorptive activity was quantified using a kit from Cosmo Bio

USA (Carlsbad, CA) and resorption pits visualized by toluidine blue staining and photographed by Nikon Eclipse TE2000-S with Q-color3 camera and Q-Capture-Pro-V7 software. Release of resorption products was quantified by fluorography using 485nm excitation and 535nm emission wavelength on a SpectraMax iD3 spectrophotometer (Molecular Devices, San Jose, CA).

**XTT Assay.** XTT assays were used to quantify cell proliferation/cytotoxicity, in response to HU (50μM) using the XTT kit of Abcam (Boston, MA) according to the manufacturer's directions. Briefly, 10,000 cells/well were seeded in a 96-well plate in DM and incubated at 37°C in 5% $CO_2$. XTT was prepared by mixing equal volumes of XTT Developer Reagent with Electron Mediator Solution and 10μL of XTT solution was added to cultures at 24hr, 48hr, 72hr and 7 days of HU treatment. After 2hr absorbance was read at 450nm on a SpectraMax iD3 spectrophotometer (Molecular Devices, San Jose, CA).

**Bromodeoxyuridine (BrdU) Proliferation assay.** To assess the effect of HU on cell proliferation we used a BrdU ELISA Kit (Abcam) according to the manufacturer's instructions. Briefly, 10,000 cells/well were seeded in 96-well plate and incubated at 37°C and 5% CO2. After 24hr, 48hr, 72hr, and 7 days of HU treatment, 1X BrdU reagent (20μL/well) was added for the last 12hr. Cells were fixed at room-temperature for 30min and incubated with anti-BrdU antibody for 1hr at RT, washed again, and incubated with peroxidase goat anti-mouse IgG for 30min at RT. Cells were washed and incubated with 3,3′,5,5′-tetramethylbenzidine (TMB) for 30min at RT before stop solution was added and optical density read at 450nm on a SpectraMax iD3 spectrophotometer (Molecular Devices).

**Apoptosis Assays.** BMSCs were plated in 12-well plates in differentiation medium (DM) comprising α-MEM supplemented with 10% FBS and 50μM/mL ascorbate and 10mM β-

glycerophosphate. Monocytes isolated from spleen were maintained in α-MEM supplemented with 10% FBS for 7-days with M-CSF (25ng/ml) from R&D Systems (Minneapolis, MN). BMSC and monocytes were treated with and without HU at 50μM and 100μM concentrations and cultured at 37°C in 5% CO2 for 7 days. Cell viability, apoptosis and necrosis was quantified using a BD Pharmingen FITC Annexin V Apoptosis Detection Kit I (BD Biosciences, San Diego, CA, USA) according to the manufacturers protocol. At the end of the experiment cells were harvested using 0.25% trypsin and washed twice with ice cold PBS before being incubated with Annexin V-FITC and propidium iodide for 15 min at room temperature in Annexin V binding buffer. Cells were analyzed by flow cytometry using an Cytek Aurora (Fremont, CA, USA). Data analysis was performed using FlowJo software V10.10.0 (Ashland, OR, USA).

**Statistical Analysis.** Significance was determined using Prizm-V10.1 for Macintosh (GraphPad Software Inc., La Jolla, CA). Gaussian distribution was assessed by Shapiro-Wilk test. Normally distributed two-sample comparisons involved unpaired two-tailed Student's t-test or Mann-Whitney test for nonparametric data. Multigroup comparisons (≥3 groups) were performed using one-way ANOVA with Tukey-Kramer post-test, or Kruskal-Wallis test with Dunn's post-test is nonparametric. $p<0.05$ was considered statistically significant and all available data are shown in graphs and used for analysis.

**Estimation of sample size for mice studies.** Given the effects of HU on bone mass in SCD and WT mice are unknown it was not possible to predict standard deviation or effect size to perform power analysis. We thus relied on published data involving previous μCT studies of Townes mice where 4-6 Townes mice/group provided statistically significant outcomes for SCD[14]. For WT mice which are readily available in larger numbers we used 9-10 mice/group

to ensure adequate power to detect small changes in response due to HU administration. The number of animals used for each group in each experiment if stated in the figure legend.

**Supplemental References**:

1. Wu LC, Sun CW, Ryan TM, Pawlik KM, Ren J, Townes TM. Correction of sickle cell disease by homologous recombination in embryonic stem cells. Blood. 2006;108(4):1183-8.

2. West GB, Brown JH. The origin of allometric scaling laws in biology from genomes to ecosystems: towards a quantitative unifying theory of biological structure and organization. J Exp Biol. 2005;208(Pt 9):1575-92.

3. Ofotokun I, Titanji K, Vikulina T, Roser-Page S, Yamaguchi M, Zayzafoon M, et al. Role of T-cell reconstitution in HIV-1 antiretroviral therapy-induced bone loss. Nat Commun. 2015;6:8282.

4. Roser-Page S, Vikulina T, Zayzafoon M, Weitzmann MN. CTLA-4Ig-induced T cell anergy promotes Wnt-10b production and bone formation in a mouse model. Arthritis Rheumatol. 2014;66(4):990-9.

5. Li Y, Toraldo G, Li A, Yang X, Zhang H, Qian WP, et al. B cells and T cells are critical for the preservation of bone homeostasis and attainment of peak bone mass in vivo. Blood. 2007;109(9):3839-48.

6. Gao Y, Wu X, Terauchi M, Li JY, Grassi F, Galley S, et al. T cells potentiate PTH-induced cortical bone loss through CD40L signaling. Cell Metab. 2008;8(2):132-45.

7. Roser-Page S, Weiss D, Vikulina T, Yu M, Pacifici R, Weitzmann MN. Cyclic Adenosine Monophosphate (cAMP)-Dependent Phosphodiesterase Inhibition Promotes Bone Anabolism Through CD8(+) T Cell Wnt-10b Production in Mice. JBMR Plus. 2022;6(7):e10636.

8. Weitzmann MN, Roser-Page S, Vikulina T, Weiss D, Hao L, Baldwin WH, et al. Reduced bone formation in males and increased bone resorption in females drive bone loss in hemophilia A mice. Blood Adv. 2019;3(3):288-300.

9. Bedi B, Li JY, Tawfeek H, Baek KH, Adams J, Vangara SS, et al. Silencing of parathyroid hormone (PTH) receptor 1 in T cells blunts the bone anabolic activity of PTH. Proc Natl Acad Sci U S A. 2012;109(12):E725-33.

10. Li YP, Chen W. Characterization of mouse cathepsin K gene, the gene promoter, and the gene expression. J Bone Miner Res. 1999;14(4):487-99.

11. Zhou X, Murphy FR, Gehdu N, Zhang J, Iredale JP, Benyon RC. Engagement of alphavbeta3 integrin regulates proliferation and apoptosis of hepatic stellate cells. J Biol Chem. 2004;279(23):23996-4006.

12. Chen D, Wang Q, Li Y, Sun P, Kuek V, Yuan J, et al. Notopterol Attenuates Estrogen Deficiency-Induced Osteoporosis via Repressing RANKL Signaling and Reactive Oxygen Species. Front Pharmacol. 2021;12:664836.

13. Monteiro LB, Davanzo GG, de Aguiar CF, Moraes-Vieira PMM. Using flow cytometry for mitochondrial assays. MethodsX. 2020;7:100938.

14. Selma J, Song H, Rivera C, Douglas S, Akella A, Bollavaram K, et al. Sickle cell disease promotes sex-dependent pathological bone loss through enhanced cathepsin proteolytic activity in mice. Blood Adv. 2022;6(5):1381-93.
